# Herbivory and pollination impact on the evolution of herbivore‐induced plasticity in defense and floral traits

**DOI:** 10.1002/evl3.200

**Published:** 2020-10-26

**Authors:** Sergio E. Ramos, Florian P. Schiestl

**Affiliations:** ^1^ Department of Systematic and Evolutionary Botany University of Zurich Zurich CH‐8008 Switzerland; ^2^ Current Address: Department of Biological Sciences University of Pittsburgh, Pittsburgh, PA, USA

**Keywords:** Experimental evolution, herbivory, phenotypic plasticity, plant defense, pollination

## Abstract

Theory predicts that herbivory should primarily determine the evolution of herbivore‐induced plasticity in plant defenses, but little is known about the influence of other interactions such as pollination. Pollinators may exert negative selection on the herbivore‐induced plasticity of chemical defenses when floral signals and rewards are indirectly affected, provoking deterrent effects on these mutualists. We investigated the influence of constant herbivory and pollination on the evolved patterns and degree of herbivore‐induced plasticity in chemical plant defenses and floral morphometry and volatiles in fast‐cycling *Brassica rapa* plants. To do this, we used plants from an evolution experiment that had evolved under bee/hand pollination and herbivory manipulated in a 2 × 2 factorial design during six generations, producing four selection treatments. We grew sibling plant pairs from each of the four selection treatments of the last generation and infested one group with herbivores and left the other uninfested. Herbivore‐induced plasticity was analyzed within‐ and between‐selection treatments. We found support for the hypothesis that constant herbivory favors the evolution of higher constitutive yet lower herbivore‐induced plasticity in defenses. However, this only occurred in plants that evolved under hand pollination and constant herbivory. Bee pollination had a strong influence on the evolution of herbivore‐induced plasticity of all traits studied. Plants that evolved under bee pollination, with and without constant herbivory, showed remarkably similar patterns of herbivore‐induced plasticity in their defense‐ and floral traits and had a higher number of plastic responses compared to plants with hand pollination. Such patterns support the hypothesis that bee pollination influenced the evolution of herbivore‐induced plasticity, most likely via indirect effects, such as links between defense‐ and floral traits. We conclude that interactions other than herbivory, such as pollination, may impact herbivore‐induced plasticity, through indirect effects and metabolic trade‐offs, when it contributes to trait evolution in plants.

**Impact Summary**

A prominent example of phenotypic plasticity in plants is changes that occur in chemical defenses and floral traits triggered by herbivory. Such herbivore‐induced plasticity has mostly been studied in terms of mechanisms and ecological consequences. Less is known about how different and contrasting interactions, such as herbivory and pollination, shape the evolution of herbivore‐induced plasticity in functionally different plant traits. Here, we used plants that had experimentally evolved under different herbivory and pollination treatments for six generations and investigated the impact of such contrasting interactions on the evolved patterns of herbivore‐induced plasticity. We show that the evolution of herbivore‐induced plasticity in the chemical defenses and floral morphology and fragrance is explained by the evolutionary history of herbivory and pollination. Our findings suggest that the type of interaction imposing the strongest selection on trait evolution may also determine the evolutionary trajectories of herbivore‐induced plasticity.

Phenotypic plasticity, the change in an organism's phenotypic characteristics in response to an environmental signal (Schlichting and Smith 2002), is thought to evolve as a mechanism to express adaptive phenotypes in variable environments and stressful conditions, allowing organisms to sustain fitness (Agrawal 1999; DeWitt et al. 1999; Levis and Pfennig 2016).

In plants, one of the most studied aspects of phenotypic plasticity is the herbivore‐induced changes in defense traits; upon herbivory, plants can upregulate the production of toxic secondary metabolites, as well as physical defenses such as trichomes (Agren and Schemske 1993; Karban and Baldwin 1997; Heil 2010). Also, the composition of leaf volatiles can change dramatically, which can be used by predators of herbivores to locate their prey and is thus interpreted as a mean of indirect defense (Dicke et al. 1990; Turlings et al. 1995; Heil 2008; Knauer et al. 2018). Because of the usually high metabolic costs of chemical and physical defenses, theory on the evolution of plant defenses predicts that variable and unpredictable herbivory should favor inducible defenses. On the other hand, constant and predictable herbivory should select for constitutive (always present) defenses (DeWitt et al. 1999; Schlichting and Smith 2002; Stamp 2003; Ito and Sakai 2009; Heil 2010; Bixenmann et al. 2016).

Previous studies have shown that insect herbivores can impose selection on plant defense traits and drive their evolution (Fornoni et al. 2004; Agrawal et al. 2012; Bode and Kessler 2012; Züst et al. 2012; Carmona and Fornoni 2013). However, the hypothesis that constant and predictable herbivory determines the extent to which defenses can evolve toward being constitutive or inducible has been experimentally less explored. Experimental evolution with *Daphnia* (Scoville and Pfrender 2010) and bacteria (Westra et al. 2015) has supported the hypothesis that constant and predictable risk of predation or attack (respectively) can favor the evolution of constitutive defenses versus inducible ones. In plants, multigenerational experiments where herbivore presence has been manipulated offer some evidence indicating that the constant presence of insect herbivores can favor the evolution of higher constitutive levels of chemical defenses (Agrawal et al. 2012; Züst et al. 2012). Comparative studies in plants have also revealed patterns that suggest that evolutionary changes from ancestral inducibility to constitutive defenses have occurred (Thaler and Karban 1997; Heil et al. 2004; Bixenmann et al. 2016). An ideal experiment to test the hypothesis that constant herbivory is the main factor determining the evolution of plasticity in defenses would be comparing plants that have evolved for several generations under constant presence or absence of herbivory.

Plastic responses to herbivory involve not only defense‐ but also floral traits, with often detrimental consequences for reproductive fitness via physiological or ecological costs (Strauss et al. 1996; Kessler and Halitschke 2009; Kessler et al. 2011; Lucas‐Barbosa et al. 2011; Barber et al. 2012; Schiestl et al. 2014; Moreira et al. 2019; Rusman et al. 2019b). Such herbivore‐induced floral plasticity can result from pleiotropic effects via resource trade‐offs, as well as the genetic, biochemical, or functional linkage between floral traits and antiherbivore defenses (reviewed in Rusman et al. 2019a). For instance, jasmonic acid (JA), a key phytohormone involved in the production of chemical and physical defense against chewing herbivores, also plays an important role in flower development; JA has been shown to affect anther elongation and pollen fertility (Stintzi and Browse 2000), style length and anthesis (Stitz et al. 2014), as well as the emission of floral volatiles (Li et al. 2018) and nectar secretion (Radhika et al. 2010). Indeed, due to the abovementioned mechanisms, recent studies have provided clear evidence that herbivores can indirectly mediate selection on floral traits and mating system (Strauss and Whittall 2006; Adler 2008; Agren et al. 2013; Sletvold et al. 2015; Santangelo et al. 2019), thus affecting their evolutionary trajectories (Ramos and Schiestl 2019, 2020).

As a flip side, pollinators can also indirectly impose negative selection on high levels of defensive leaf compounds. Such negative selection can be possible because the induction of defenses in vegetative tissues can lead to their accumulation in floral nectar and pollen (Strauss et al. 2004; Adler et al. 2012; Palmer‐Young et al. 2019; Ramos and Schiestl 2019), or provoke a deterrent effect in flower fragrance (e.g., due to increased emission of terpenoids; Kessler and Halitschke 2009; Kessler et al. 2011). Also, studies showing that transitions in plant mating system (e.g., from outcrossing to selfing) can occur with concomitant changes in the defense strategy offer another line of evidence of the non‐independent evolution of pollination and plant defenses (Campbell and Kessler 2013; Campbell et al. 2013; Johnson et al. 2015).

Taken together, the link between pollination‐ and defense‐related traits points out the need to integrate pollination as an additional factor in the plant defense theory (Heath et al. 2014; Johnson et al. 2015). Here, we target this topic by analyzing the patterns of herbivore‐induced plasticity in defenses (glucosinolates in leaves), and floral traits (morphology and volatiles) in *Brassica rapa* plants. These plants had evolved for six generations under herbivory and hand/bee pollination manipulated in a full factorial design, resulting in four selection treatments with different evolutionary history (Fig. 1) (Ramos and Schiestl 2019). We focused on the analysis of plasticity, considering it could have evolved toward different trajectories according to the selective pressures imposed during our previous experimental evolution study. Thus, to reveal the evolved levels of plasticity of the traits inspected, we used the seeds of the last generation of our previous experiment (i.e., the eighth generation; see Ramos and Schiestl 2019), and regrew pairs of sibling plants from each selection treatment and replicate. We exposed one sibling to herbivory by *Pieris brassicae* (infested treatment), whereas the other sibling was left uninfested (noninfested treatment) (Fig. 1). With this simple experimental design, we specifically addressed the hypothesis that constant herbivory (i.e., over six generations) is the most important factor in determining the evolution of plasticity of plant defenses, favoring high constitutive levels of leaf glucosinolates (thus, reduced plasticity), regardless of the pollination history. If so, we should find that plants that evolved for six generations under constant herbivory, and either bee (B_H_+) or hand pollination (H_H_+), should show less plasticity in defenses and flower traits than plants that evolved without herbivory (i.e., B_H_– and H_H_–). This would indicate that the history of pollination has no influence on the evolution of plasticity in defense and flowers. Additionally, as a nonmutually exclusive hypothesis, we could expect that the evolutionary history of pollination had an influence on the evolution of plasticity of defenses and flowers beyond the sole effect of constant herbivory. Specifically, bee pollination could indirectly influence the evolution of plastic defenses when selecting for floral attractiveness and nontoxic rewards (Adler et al. 2012). If so, we should find that plants that evolved under bee pollination, both with constant herbivory (B_H_+) or without (B_H_–), should show higher plasticity levels in defense and floral traits compared to plants of hand pollination (H_H_+ and H_H_–). Following univariate and multivariate approaches, we performed a detailed analysis of plasticity at the within‐ and between‐selection treatment level. We also tested the influence that pollination, herbivory, and their interaction during experimental evolution may have had on the evolution of the plasticity for all plant traits studied. Additionally, we explored the relationship of the mean trait values and their degree of reaction norm to examine possible associations between trait evolution and plasticity.

**Figure 1:**
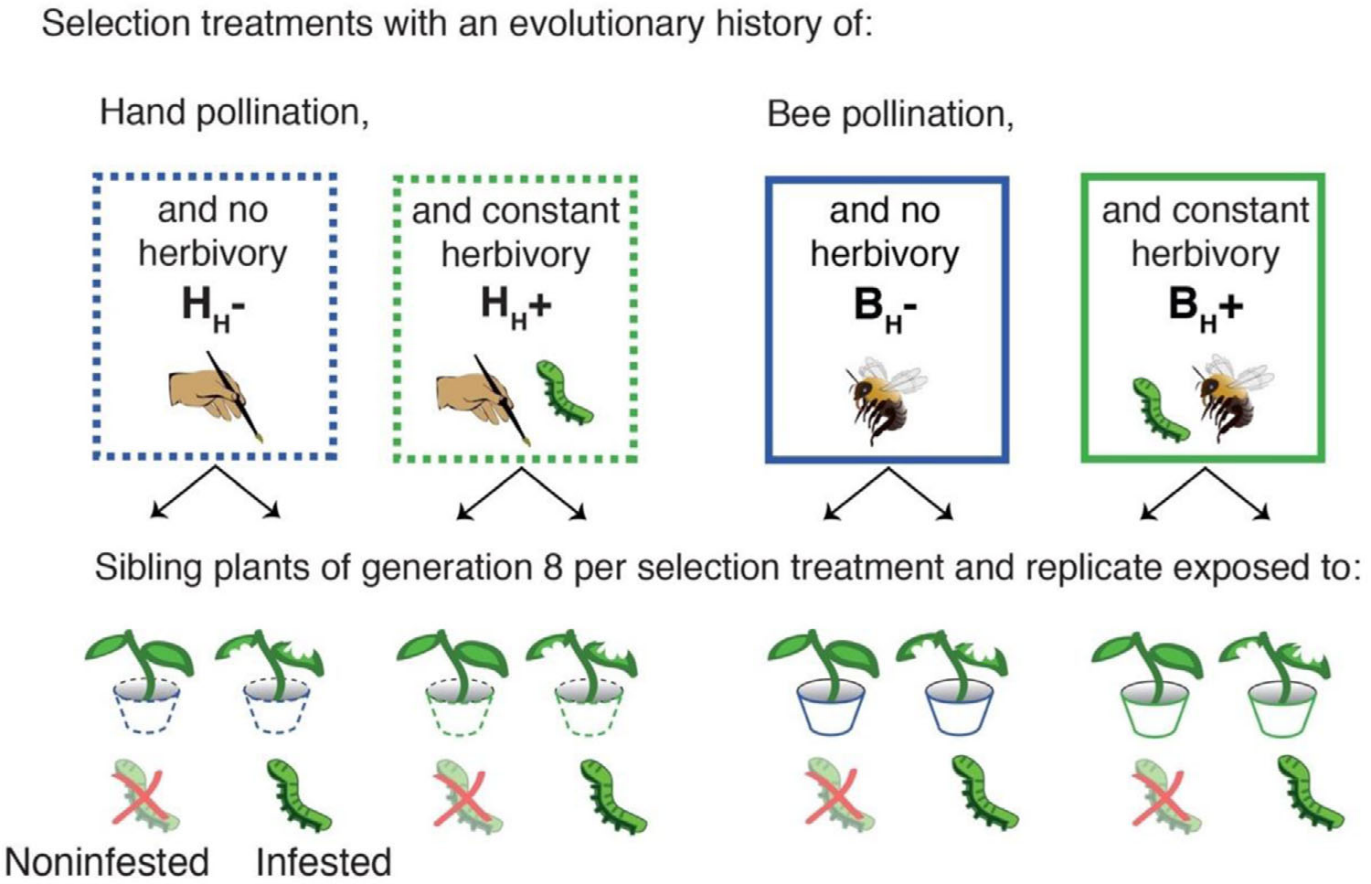
Experimental design of our study. In our coding of the selection treatments, the first letter refers to the pollination history (H for hand pollination and B for bee pollination), whereas the subscript _H_ indicates herbivory history, with a – symbol indicating no herbivory history and a + symbol indicating a history of constant herbivory. Sibling plants from mothers with different evolutionary history of selection were exposed to one of two herbivory environments: noninfested and infested with *Pieris brassica* caterpillars (see Methods). Thus, for plants in the selection treatments of H_H_– and B_H_– infestation represented a novel environment after experimental evolution. Each selection treatment was replicated three times (see Methods for details).

## Results

### PLASTICITY COMPARISONS IN HERBIVORE‐INDUCED DEFENSE AND FLORAL TRAITS

Differences in the degree of plasticity of all plant traits were analyzed following two approaches, namely, (i) within‐ and (ii) between‐selection treatments. For (i), we found that one to five leaf glucosinolates were plastic across selection treatments, all of them increasing in concentration upon infestation (Tables 1 and S1). Plants that evolved under bee pollination and no herbivory (B_H_–) showed the highest number of plastic glucosinolates, whereas only one leaf glucosinolate was plastic in plants that evolved under hand pollination and constant herbivory (H_H_+) (Table 1). In contrast to the glucosinolates, all floral traits (morphological and volatiles) decreased on average upon infestation; the number of plastic traits was also variable across selection treatments (Tables 1 and S1). These responses in the flowers were judged as pleiotropic responses to the plasticity of the direct defense (glucosinolates). As a general pattern, plants with an evolutionary history of bee pollination without herbivory (B_H_–) showed pleiotropic responses in the most traits (10 traits; Table 1). In the other selection treatments, three to five morphological floral traits showed pleiotropic responses; in plants of B_H_– and B_H_+, the same five floral traits were plastic, whereas in H_H_– and H_H_+ only petal length and width were plastic traits (Table 1). Among floral volatiles, five out of 13 floral volatiles—mostly aromatic compounds—showed a decrease in at least one selection treatment upon infestation, except in H_H_– plants were none responded (Table 1). In B_H_– plants, five floral volatiles showed a plastic response, whereas only one volatile responded in H_H_+ and B_H_+ plants.

**Table 1:** Plasticity comparisons (noninfested and infested plants) within past selection treatments (pollination, herbivory) in 28 plant traits. Values show the mean ± SD. Numbers in bold indicate significant differences between noninfested and infested sibling plants (*P* < 0.05; see Table S1 for statistics). Arrows that point up (↑) indicate an increase upon infestation and arrows that point down (↓) indicate a decrease upon infestation. Leaf glucosinolates are in μg mL^−1^ 100 mg leaf tissue, morphometric traits are in mm, and floral volatiles are in pg flower^– 1^ L^−1^. *N* = sample size by selection treatment. Numbers preceding the trait names are used as a trait ID for Figure 3

	Hand pollination							Bee pollination				
		No herbivory (H_H_–)			Herbivory (H_H+_)			No herbivory (B_H_–)			Herbivory (B_H+_)	
Trait		Noninfested	Infested		Noninfested	Infested		Noninfested	Infested		Noninfested	Infested
Leaf glucosinolates		*N =* 52			*N* = 49			*N* = 55			*N* = 54	
1. Glucobrassicanapin^a^	↑	**59.01 ± 28.62**	**119.17 ± 78.95**		69.85 ± 57.35	104.02 ± 70.08	↑	**91.52 ± 41.13**	**214.58 ± 149.83**	↑	**97.89 ± 55.05**	**218.12 ± 132.43**
2. Glucoerucin^a^		1.15 ± 2.24	0.88 ± 1.65		0.21 ± 0.23	0.25 ± 0.32	↑	**0.24 ± 0.26**	**0.3 ± 0.36**		0.29 ± 0.25	0.32 ± 0.26
3. Gluconapin^a^	↑	**2311.11 ± 1116.91**	**3940.21 ± 1584.9**		2681.38 ± 1811.66	3591.67 ± 1355.86	↑	**2663.57 ± 1274.51**	**4645.73 ± 1961.46**	↑	**2558.76 ± 1328.8**	**4348.87 ± 1894.94**
4. Gluconasturtiin^a^		43.76 ± 61.75	27.17 ± 31.11		49.17 ± 64.54	37.05 ± 54.05		49.8 ± 49.31	68.5 ± 47.86		48.02 ± 61.32	51.85 ± 57.71
5. Glucoraphanin^a^		7.49 ± 8.48	7.28 ± 6.93		5.62 ± 5.9	7.1 ± 8.86	↑	**4.65 ± 3.94**	**7.78 ± 8.45**		8.82 ± 8.02	10.18 ± 8.82
6. Glucobrassicin^b^		35.07 ± 29.74	22.9 ± 11.68		48.32 ± 37.53	49.04 ± 33.47		39.77 ± 28.07	40.84 ± 31.82		35.8 ± 17.53	38.64 ± 31.28
7. Hydroxyglucobrassicin^b^		2.13 ± 2.1	1.22 ± 0.8		2.77 ± 2.56	3.16 ± 2.6		2.02 ± 1.62	2.13 ± 2.26		2.13 ± 1.09	2.23 ± 1.95
8. Methoxyglucobrassicin^b^		10.97 ± 8.26	7.32 ± 5.46		30.84 ± 32.98	28.64 ± 18.74		14.87 ± 16.15	14 ± 13.61		19.23 ± 19.66	17.5 ± 13.86
9. Neoglucobrassicin^b^	↑	**13.37 ± 2.04**	**42.09 ± 39.69**	↑	**12.54 ± 1.23**	**42.98 ± 33.28**	↑	**14.1 ± 2.84**	**57.26 ± 110.29**	↑	**15.39 ± 4.91**	**59.36 ± 109.54**
Flower morphometry		*N* = 72			*N* = 67			*N* = 74			*N* = 86	
10. Sepal length		5.28 ± 0.57	5.1 ± 0.44	↓	**5.23 ± 0.44**	**4.92 ± 0.37**	↓	**5.58 ± 0.4**	**5.19 ± 0.49**	↓	**5.12 ± 0.51**	**4.78 ± 0.47**
11. Petal length	↓	**5.97 ± 0.83**	**5.63 ± 0.68**	↓	**6.11 ± 0.57**	**5.68 ± 0.57**	↓	**6.41 ± 0.6**	**5.8 ± 0.67**	↓	**5.93 ± 0.62**	**5.72 ± 0.58**
12. Petal width	↓	**4.57 ± 0.56**	**4.26 ± 0.43**	↓	**4.65 ± 0.53**	**4.22 ± 0.52**	↓	**5.01 ± 0.58**	**4.41 ± 0.68**	↓	**4.75 ± 0.52**	**4.41 ± 0.61**
13. Pistil length		8.21 ± 0.98	7.83 ± 0.87	↓	**7.35 ± 0.65**	**6.9 ± 0.65**		7.73 ± 0.81	7.2 ± 0.84		7.11 ± 0.81	7.07 ± 0.78
14. Long stamen	↓	**7.43 ± 0.65**	**7.25 ± 0.6**		7.38 ± 0.41	7.13 ± 0.52	↓	**7.46 ± 0.47**	**7.22 ± 0.65**	↓	**7.2 ± 0.66**	**7.17 ± 0.51**
15. Short stamen		5.15 ± 0.73	4.87 ± 0.57		5.27 ± 0.85	5.13 ± 0.74	↓	**5.19 ± 0.65**	**4.96 ± 0.55**	↓	**4.99 ± 0.67**	**4.8 ± 0.51**
Flower volatiles		*N* = 40			*N* = 40			*N* = 42			*N* = 51	
16. Benzaldehyde		602.41 ± 269.56	721.7 ± 387.67		595.9 ± 276.89	671.79 ± 362.74		702.44 ± 329.38	739.57 ± 410.4		615.44 ± 361.94	642.84 ± 320.36
17. 1‐Butene‐4‐isothyocyanate		53.5 ± 54.01	79.72 ± 67.04		107.6 ± 135.88	93.98 ± 75.74		57.08 ± 92.69	69.5 ± 64.83		82.56 ± 75.44	64.74 ± 59.09
18. 6‐Methyl‐5‐hepten‐2‐one		64.36 ± 34.16	78.83 ± 43.61		70.65 ± 55.77	73.67 ± 35.83		83.37 ± 39.12	83.06 ± 37.66		80.2 ± 35.51	87.87 ± 38.7
19. (*Z*)‐3‐Hexenyl acetate		36.59 ± 20.57	43.67 ± 22.1		45.47 ± 32.7	44.39 ± 23.35	↓	**57.35 ± 45.46**	**35.99 ± 30.77**		24.61 ± 13.08	36.06 ± 33.41
20. Phenylacetaldehyde		58.58 ± 134.72	28.31 ± 35.07		95.06 ± 146.52	61.91 ± 78.29	↓	**120.1 ± 163.17**	**41.13 ± 61.69**	↓	**88.31 ± 194.88**	**25.46 ± 22.6**
21. Methyl benzoate		100.66 ± 86.05	70.94 ± 48.59		168.74 ± 288.3	76.97 ± 56.16		120.51 ± 98.93	65.12 ± 42.77		94.35 ± 90.97	80.65 ± 59.58
22. Benzyl nitrile		65.14 ± 62.3	35.57 ± 23.17		99.21 ± 111.65	68.8 ± 46.03	↓	**159.25 ± 149.98**	**66.18 ± 67.07**		70.02 ± 56.03	40.85 ± 45.88
23. Methyl salicylate		41.73 ± 26.28	38.82 ± 31.37		31.98 ± 24.89	37.96 ± 38.08		34.41 ± 24.45	32.56 ± 28.66		39.51 ± 29.85	32.56 ± 28.53
24. 2‐Aminobenzaldehyde		453.3 ± 853.67	314.13 ± 352.48	↓	**375.14 ± 479.72**	**200.65 ± 204.83**		595.59 ± 654.23	213.41 ± 267.32		500.32 ± 631.8	371.79 ± 670.17
25. *p*‐Anisaldehyde		13.23 ± 20.48	8.24 ± 12.58		18.13 ± 27.33	17.34 ± 24.02		37.46 ± 32.74	28.11 ± 26.49		18.37 ± 20.73	15.59 ± 23.86
26. Indole		126.56 ± 145.45	72.52 ± 69.28		113.31 ± 108.73	82.84 ± 74.69	↓	**193.24 ± 167.59**	**82.41 ± 88.18**		152.43 ± 151.44	89.66 ± 111.79
27. Methyl anthranilate		257.93 ± 316.27	167.25 ± 162.5		332.62 ± 406.6	483 ± 1160.05		265.71 ± 200.45	155.14 ± 154.28		199.79 ± 202.47	168.59 ± 218.92
28. (*E,E*)‐α‐Farnesene		1296.97 ± 812.29	1165.33 ± 624.78		683.53 ± 470.06	539.96 ± 478.51	↓	**1904.43 ± 1530.6**	**1145.65 ± 935.82**		1792.98 ± 905.35	1332.26 ± 701.34
Number of plastic traits		**6**			**6**			**15**			**9**	

^a^Aliphatic glucosinolates.

^b^Indolic glucosinolates.

**Figure 2:**
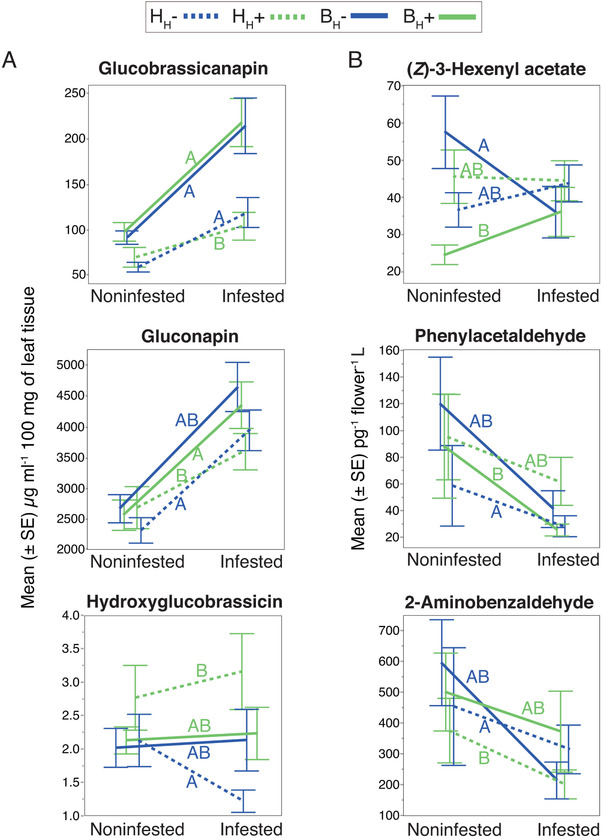
Plasticity comparisons between selection treatments. Only six plant traits showed differences in their herbivore‐induced plasticity. (A) Three leaf glucosinolates and (B) three floral volatiles. Different letters on each connecting line indicate significant differences between treatments through comparisons of the sibling reaction norm (HSD Tukey post hoc test after LMMs per trait, *P* < 0.05; FDR adjusted *P*‐values used for multiple comparisons between selection treatments; see Table S2 for full statistics and sample sizes).

For comparisons between selection treatments, we used the sibling reaction norm values obtained from the subtraction of infested from noninfested values for each trait at the sibling plant‐pair level (see details in methods). From the univariate analysis, we detected differences in the reaction norm between treatments for three glucosinolates and three floral volatiles (Fig. 2).

For glucobrassicanapin, plants that evolved under bee pollination, with (B_H_+) and without herbivory (B_H_–), and those that evolved under hand pollination without herbivory (H_H_–) showed more inducibility compared to plants of hand pollination and herbivory (H_H_+) (Fig. 2A). The constitutive amount of this glucosinolate was marginally significantly higher in plants that evolved under bee pollination compared to those with hand pollination (linear mixed model [LMM], treatment: *F*_3,6_ = 3.96, *P* = 0.06), but the values for infested plants were not different (*P* = 0.455). For gluconapin, plants that evolved under bee pollination, with (B_H_+) and without herbivory (B_H_–), showed a similar degree of plasticity, but plants of B_H_+ and H_H_– differed from plants that evolved under hand pollination and herbivory (H_H_+) (Fig. 2A). For hydroxyglucobrassicin, plasticity was different only between plants that evolved under hand pollination, with (H_H_+) and without herbivory (H_H_–) (Fig. 2A). Interestingly, glucobrassicanapin and gluconapin showed the expected pattern of lower inducible responses in plants that evolved under hand pollination and constant herbivory (H_H_+).

For the three floral volatiles, we observed that plants that evolved under bee pollination without herbivory (B_H_–) showed more intense plastic responses of decrease upon infestation. In contrast, plants from the other selection treatments showed variable patterns in their plastic responses (Fig. 2B; Table S2).

**Figure 3:**
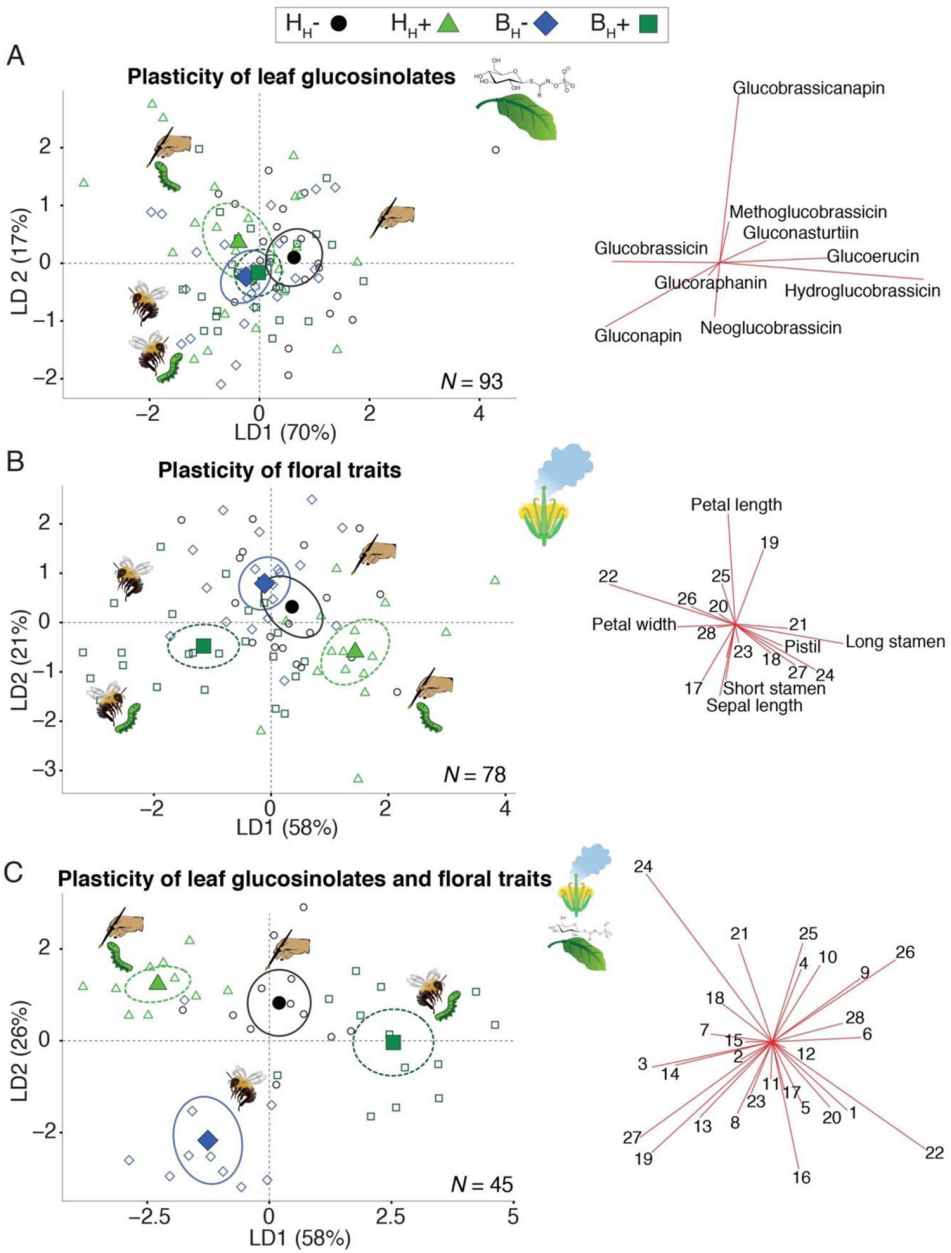
Multivariate comparisons of plasticity between selection treatments. Each dot represents a sibling reaction norm calculated as the difference of noninfested and infested values per sibling plant pairs. Enlarged symbols indicate group centroids and are enclosed by 95% confidence ellipses. Numbers in the corresponding biplots indicate trait ID following Table 1. The origin of the biplot vectors starts at (0,0), but is shifted for clarity in each graph. Analyses were predefined by selection treatment. (A) Linear discriminant analysis (LDA) performed using the nine leaf glucosinolates. The LDA shows a trend indicating that the plasticity in the defenses is more similar between plants with a history of bee pollination with (B_H_+) and without (B_H_–) constant herbivory than the other selection treatments. (B) LDA performed only with the 19 floral traits. This analysis shows different evolutionary trajectories in the plasticity of flowers as a result of their evolutionary history of bee and hand pollination and constant herbivory. (C) LDA combining the plasticity of 28 defense and floral traits. The analysis shows an even more clear pattern of variation in the plasticity between the four selection treatments owing to their evolutionary history of pollination and herbivory. *N* = sample size per LDA.

From multivariate analyses via linear discriminant analyses (LDAs) using only the nine leaf glucosinolates, we found a trend suggesting that plants that evolved under bee pollination, with (B_H_+) and without herbivory (B_H_–), evolved similar levels of herbivore‐induced plasticity in their defenses (Fig. 3A). It also showed a trend that suggests that plants that evolved under hand pollination and herbivory (H_H_+) evolved toward a different evolutionary trajectory in the plasticity of their defenses compared to the other selection treatments (Fig. 3A). Together, these results point out that a history of pollination by bees and their direct selection on floral traits was an important factor in indirectly determining the evolution of plasticity of defenses.

An LDA using only the floral traits (19 traits combining morphology and volatiles) showed a clear pattern of different evolutionary trajectories in the plasticity of floral traits between plants with constant herbivory, either with bee (B_H_+) or hand pollination (H_H_+) and between the plants without herbivory (B_H_– and H_H_–) (Fig. 3B). Such a pattern of different evolutionary trajectories in the herbivore‐induced plasticity between the four selection treatments became clearer when leaf glucosinolates and floral traits were combined (Fig. 3C). These results point out that an evolutionary history of both herbivory and pollination, alone and in combination, influenced the evolved levels of herbivore‐induced plasticity of plant traits.

### 1 THE INFLUENCE OF POLLINATION, HERBIVORY, AND THEIR INTERACTION ON THE EVOLVED PATTERNS OF PLASTICITY

We tested the effects of pollination, herbivory, and their interaction (P × H) on sibling reaction norms with multivariate and univariate approaches. For the multivariate approach, we used the resulting linear discriminant functions from the above‐described LDAs using (i) only leaf glucosinolates, (ii) floral traits, and (iii) leaf glucosinolates and floral traits combined and performed LMMs. For (i), we found that pollination had a significant effect on discriminant function 2, whereas the P × H interaction affected discriminant function 1 (see trait contributions in biplot; Fig. 3A). Interestingly, the factor herbivory had no effect on any discriminant function (Table S3). For (ii), we found an effect of pollination and the P × H interaction on discriminant functions 1 and 3, and an effect of herbivory on discriminant function 2 (Table S3; Fig. 3B). For (iii), we found an effect of pollination and the P × H interaction on discriminant function 1, an effect of the three factors on discriminant function 2, and an effect of herbivory on discriminant function 3 (Table S3; Fig. 3C).

From the univariate analyses, we found that pollination had an effect on the aromatic volatiles phenylacetaldehyde and benzyl nitrile, the P × H interaction affected hydroxyglucobrassicin and petal length, and herbivory had no effect on any trait (Table S4). The results of these multivariate and univariate analyses indicated that the evolution of the herbivore‐induced plasticity was mostly affected by the mode of pollination and the interaction of pollination and herbivory.

### 2 CORRELATION OF MEAN TRAIT VALUE AND MEAN REACTION NORM

**Figure 4:**
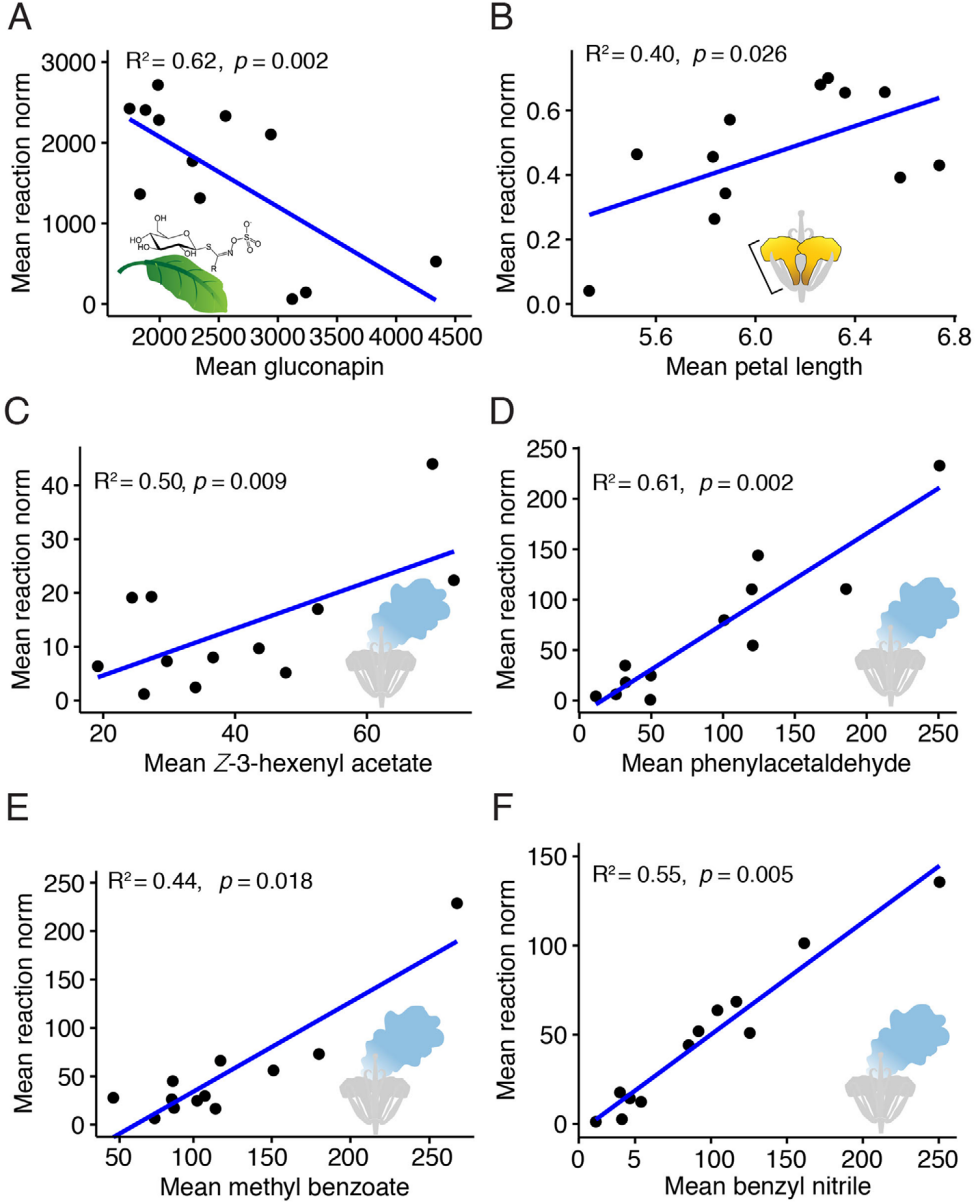
Pearson correlations between mean trait values (calculated for each replicate) and the mean reaction norm (absolute values) for different plant traits. Each dot is the mean per replicate per treatment (four selection treatments with three replicates each = 12 replicates). Determination coefficients correspond to correlations performed with ln transformed data. In each plot, the *x*‐ and *y*‐axes are displayed in the corresponding units of each variable: (A) gluconapin μg mL^–1^ 100 mg leaf tissue; (B) petal length mm; (C‐F) floral volatiles pg flower^−1^ L^−1^. (A) The negative correlation in gluconapin indicates that the degree of plastic response (increase) upon herbivory tends to be lower in plants with higher constitutive levels. (B‐F) The positive correlations in the floral traits indicate a higher plastic response (decrease) upon herbivory tends to be higher with higher mean trait values.

Of nine glucosinolates analyzed, only gluconapin showed a correlation between mean trait value and mean sibling reaction norm; this correlation was negative, suggesting a reduced plastic response upon herbivory at higher constitutive levels (Fig. 4A). Among floral traits, one morphological trait and four floral volatiles showed significant correlations, all of them positive, suggesting an increased plastic response upon herbivory at higher mean trait values (Fig. 4B‐F).

## Discussion

Theory on the evolution of plant defenses and their plasticity has been centered on the impact of herbivores (Mauricio and Rausher 1997; Stamp 2003). However, recent discussions have pointed out the need for a more holistic approach that takes into account the indirect influence of other biotic interactions and plasticity in other traits that may be correlated to defense traits (Poelman et al. 2008; Heath et al. 2014). In line with this view, in this study, we investigated the role of constant herbivory and pollination on the degree of herbivore‐induced plasticity of a broad range of plant traits that mediate interactions with herbivores and pollinators. We found support for the hypothesis that constant herbivory influences the evolution of plasticity in glucosinolates and floral traits, specifically by reducing the degree of plasticity. Furthermore, and more surprisingly, we found that the mode of pollination, as well as interactions between pollination and herbivory, strongly influenced the evolution of herbivore‐induced plasticity of all plant traits studied. Thus, these findings support the hypothesis that plasticity evolves in response to various kinds of interactions, most likely via direct and indirect effects.

### 1 POLLINATION IS IMPORTANT IN THE EVOLUTION OF HERBIVORE‐INDUCED PLASTICITY

So far, few studies have considered the role of pollination as a potentially important factor for the evolution of defense strategies (Herrera et al. 2002; Strauss et al. 2004; Kessler and Halitschke 2009; Kessler et al. 2011; Adler et al. 2012; Campbell and Kessler 2013; Ramos and Schiestl 2019). Moreover, we are not aware of a study that has explicitly evaluated the influence of pollination on the evolution of herbivore‐induced plasticity in plant defenses and floral traits. Pollinators may select against defense compounds because of their deterrent effects (Kessler and Halitschke 2009). For example, in a comparative analysis of several *Nicotiana* species, Adler et al. (2012) showed that those species that are highly pollinator dependent had the lowest nicotine levels in floral nectar, flower parts, and leaves. The study of Adler et al. (2012) provided evidence that pollinator‐mediated selection on floral traits and rewards could limit the evolution of chemical plant defenses, presumably through negative selection due to pollinator deterrence.

In our study, the effect of constant herbivory on the evolved degree of plasticity of leaf glucosinolates was evident only for plants with an evolutionary history of hand pollination and herbivory (H_H_+). These plants showed higher constitutive concentrations of (noninfested) gluconapin and hydroxyglucobrassicin, and reduced plastic responses to herbivory (Fig. 2A, dotted green lines). This finding nicely supports the hypothesis that a history of constant herbivory selects for constitutive defense so that defense is already in place when herbivores attack (Bixenmann et al. 2016). More surprisingly, we also found that the mode of pollination had an influence on plasticity in glucosinolates. Most likely, bees select for various patterns of plasticity in glucosinolates through the presence of these compounds in floral nectar (Ramos and Schiestl 2019). Also, metabolic links between glucosinolates and floral traits may impact the detected patterns.

For floral traits, it is well known that herbivory triggers plastic responses, typically a decrease, which is usually interpreted as pleiotropic, nonadaptive effects, because of their negative consequences on pollinator attraction and thus plant reproductive fitness (Strauss et al. 2004; Kessler et al. 2011; Lucas‐Barbosa et al. 2011; Bruinsma et al. 2014; Schiestl et al. 2014). However, recent studies have suggested that the plastic reduction of floral signals triggered by herbivory—specifically volatiles—might be an adaptive strategy to avoid interference with leaf volatiles, which are important for attracting predators of herbivores (Schiestl et al. 2014; Desurmont et al. 2020). Our results provide evidence that the evolution of herbivore‐induced plasticity in flowers is mainly influenced by pollination and the P × H interaction, and to a lesser extent, by herbivory (Table S3).

### 2 ASSOCIATION BETWEEN MEAN TRAIT VALUE AND MEAN REACTION NORM

Our finding of a correlation between the mean trait value and the reaction norm in some traits suggests that herbivore‐induced plasticity can be dependent on trait values. The negative correlation of gluconapin (Fig. 4A), indicating lower levels of plasticity (an increase) at higher concentrations, supports the prediction of the optimal defense theory that plasticity should decrease when the constitutive defense is high. This association, however, may also simply be the consequence of the metabolic costs of synthesizing glucosinolates. Thus, the increase of glucosinolates after herbivory may be less when these defense compounds are produced at higher constitutive levels, because of an upper limit of metabolic investment. Such a trade‐off between constitutive and induced concentrations of glucosinolates nicely fits with the observed patterns of a meta‐analysis of trade‐offs in plant defenses against herbivores (Koricheva et al. 2004).

On the other hand, floral traits showed positive correlations between mean trait values and their plasticity (decrease), indicating that plants with high trait values showed stronger reductions compared to those with lower values. Again, this can be explained by metabolic cost trade‐offs, as trait reduction after herbivory is usually interpreted as reallocation of resources to defense (Lucas‐Barbosa 2016). Following this logic, plants with larger floral displays invest more resources into flowers and thus tend to reallocate more upon herbivory, leading to stronger decreases in floral traits.

The link between trait values and their plasticity also offers a mechanistic explanation as to why pollination was a more important predictor in the evolution of plasticity than herbivory. Pollination mode, specifically bee pollination, was the most influential evolutionary factor in our previous experimental evolution study, which yielded the plants used here (Ramos and Schiestl 2019), and bee pollination selected for larger and more fragrant flowers. As a consequence, these plants also showed the strongest pattern of plasticity in floral traits.

## Conclusion

Our results demonstrate yet another link between pollination and herbivory, namely, through the evolution of herbivore‐induced plasticity. Cross talk between defense‐ and pollination‐related traits is likely a generally important factor determining herbivore‐induced plasticity in plants. An even more enhanced understanding of how microevolutionary processes result in plasticity and how plasticity influences trait evolution will in the future be achieved by studying the combined effect of different biotic and abiotic interactions.

## Methods

### STUDY SYSTEM AND EXPERIMENTAL EVOLUTION SET UP

The experiments carried out in this study were done using plants of the last generation from the study of Ramos and Schiestl (2019); therefore, we briefly describe here the selection treatments used in that study. These plants (fast cycling *Brassica rapa*, obtained initially from Carolina Biological Supply Co., Burlington, VT) have been exposed during six generations to one of four different selection treatments, with hand pollination, bee pollination, and presence/absence of herbivory manipulated in a 2 × 2 factorial design. Thus, the resulting selection treatments were (i) hand pollination and no herbivory (H_H_–), (ii) hand pollination and constant herbivory (H_H_+), (iii) bee pollination and no herbivory (B_H_–), and (iv) bee pollination and constant herbivory (B_H_+). Note that in our coding of these treatments, the first letter refers to the pollination history (H for hand pollination, and B for bee pollination), whereas the subscript _H_ indicates herbivory history, with a – symbol indicating no herbivory history and a + symbol indicating a history of constant herbivory (Figs. 1 and S1). During the experimental evolution, the selection treatments were replicated three times each generation (replicates A, B, and C), with each replicate sowed out sequentially in the same growth chamber (phytotron). In generations 7 and 8, insects were no longer applied, and plants of generation 7 were crossed between replicates within treatments to overcome inbreeding effects; plants of the subsequent generation 8 were used in this study. Bumblebees (*Bombus terrestris*, Andermatt Biocontrol, Grossdietwil, Switzerland) were used as pollinators. The hives were kept in a flight cage (3 m × 1 m × 1 m) inside the greenhouse. Bumblebees were fed with supplemental pollen (Biorex, Ebnat‐Kappel, Switzerland) and sugar water, as well as with *Brassica rapa* flowers. *Pieris brassicae* caterpillars were used as herbivores and applied to preflowering plants for 72 h on day 14 after seed sowing out. The caterpillars were obtained from an in‐house rearing; a detailed description of the rearing conditions can be found in Knauer and Schiestl (2017).

In this study, we considered a family as the seeds produced by a mother plant crossed with one randomly chosen father in generation 7 in each selection treatment (for more details, please refer to Ramos and Schiestl 2019). Because we kept the number of individual plants constant at 36 per replicate per selection treatment over the generations (Ramos and Schiestl 2019), we thus had a maximum of 36 families available per replicate per selection treatment at generation 8. Nevertheless, due to seed availability, we sowed out five seeds from 20 to 25 families per replicate and selection treatment in three cohorts (one cohort representing one replicate) with a 1‐week difference between them. We then randomly choose two full‐sib plants per family for our experiments.

### 1 PHENOTYPIC PLASTICITY OF LEAF GLUCOSINOLATES AND FLORAL TRAITS

To evaluate evolutionary changes in the plasticity of leaf glucosinolates and floral traits in plants of generation 8, we assigned one of the two sibling plants to either no herbivory (i.e., noninfested plants) or herbivory (i.e., infested plants; Fig. 1). Infested plants were infested with two first‐second instars of *P. brassicae* caterpillars for 48 h starting on day 10 after sowing out. For infestation, each caterpillar was placed separately on the two biggest leaves of each plant. Leaf consumption by the caterpillars was monitored twice a day to check for feeding activity and to visually inspect that the leaf tissue was approximately equally damaged in all plants (damage was not quantitatively assessed). Caterpillars with low or no feeding activity were immediately replaced by a new first or second instar one. Noninfested plants did not get in contact with *Pieris* caterpillars and separated from herbivore‐infested plants by at least 20 cm (distance between trays). All plants were sowed out and grown in a phytotron in individual pots (7 × 7 × 8 cm^3^) using standardized soil (Einheitserde, classic, EinheitserdeWerkverband e.V., Germany), kept under 24 h light, 21°C, and 60% humidity, and watered twice a day (at 0800h and 1800h). Plants were kept in the phytotron until day 22 after sowing out and afterward were transferred to an air‐conditioned greenhouse at 23°C with additional illumination.

### 2 PLANT TRAITS

All plant trait measurements of sibling plant pairs were done at the same time to minimize variation due to temporal changes and plant development. For leaf glucosinolates, around 100 mg of fresh leaf tissue from three to 17 plants of each herbivore environment (noninfested and infested), replicate, and selection treatment was collected on day 25 after sowing out. Nine glucosinolates were identified in our leaf samples, and all were considered for statistical analyses. The total sample size was *N* = 210 samples. Additional information on chemical quantification can be found in the Supporting Information. For floral traits, six morphological traits (Sepal length; petal length and width; pistil length; and long and short stamen length) were measured in three fully open flowers per plant with a digital caliper of the nearest 0.01 mm (Toolcraft, Japan). These morphometric floral traits were measured on day 23 after sowing out. For subsequent statistical analyses, the mean of each floral trait per plant was calculated. The total sample size was *N* = 299 samples.

Flower volatiles were collected on days 21 and 22 by headspace sorption from a whole inflorescence per plant with at least five opened flowers, using a push‐pull system (Ramos and Schiestl 2019). Inflorescences were carefully inserted in glass cylinders previously coated with sigmacote (Sigma‐Aldrich) and closed with a Teflon plate. Air from the surrounding was pushed with a flow rate of 100 mL min^–1^ through activated charcoal filters into the glass cylinder. Simultaneously, the air was pulled from the glass cylinder with a flow rate of 150 mL min^–1^ through a glass tube filled with ∼30 mg Tenax TA (Supelco, Bellefonte, USA). Air from empty glass cylinders was collected as air controls. The matrix included 13 flower volatiles and a total sample size of *N* = 173 samples. Leaf volatiles from infested plants were collected during 2 h on day 12 after sowing out using the same push‐pull system used for floral volatiles; caterpillars were removed from the plants the previous night. For sampling, each plant was introduced into a glass cylinder with the soil covered with aluminum plates made out of aluminum foil with a hole in the center, leaving out only the stem. This procedure intended to reduce volatiles emitted by the soil. Control samples consisted of collecting air from a glass cylinder with a pot filled with soil and covered with an aluminum plate with a hole in the center. Floral and leaf volatiles were collected in a phytotron under the standardized light and temperature conditions mentioned above. The matrix included 21 leaf volatiles and a total sample size of *N* = 174 samples. Additional information on floral and leaf volatile quantification can be found in the Supporting Information.

### 3 PARASITOID PREFERENCES

With the infested plants from the four selection treatments, we performed bioassays to test for parasitoid preferences on days 13 and 14 after sowing out (before the onset of flowering). At the time of the bioassays, all plants had their herbivores removed on the previous day, as leaf scent collection was done on day 12. Bioassays were done by replicate (A, B, and C, three cohorts) with 1 week of difference between each other. We used a six‐arm olfactometer (Turlings et al. 1995) with six cylinders made out of glass previously coated with sigmacote (Sigma‐Aldrich). One plant per treatment (i.e., one set of four plants) was randomly placed in a clean six‐arm olfactometer. The two remaining cylinders were filled with either a pot with soil or with an empty pot. Clean air was pushed through each cylinder with a flow rate of 0.7 L min^−1^. The pushed air converged into a central chamber, in which five mated female *Cotesia glomerata* parasitoid wasps were released. All wasps were obtained from an in‐house rearing. The wasps could fly toward each of the cylinders, where they were trapped in an associated glass vessel. After 30 min, the wasps in the vessels were counted and removed; wasps residing in the central chamber were counted as undecided. This procedure was repeated 12 times, each one consisting of a release of five wasps to the same set of plants. After each bioassay, the olfactometer was cleaned with acetone and dried in a ventilated oven at 80°C for 60 min.

### 4 STATISTICAL ANALYSIS

Although variation in phenotypic plasticity is usually estimated from variation among individual genotypes or populations, our approach compared unique full‐sib pairs pertaining to three replicates for each of the four treatments of evolutionary history (cf. Teotonio and Rose 2009). Hence, depending on the analysis, we took replicates (three per treatment) or the selection treatments as our units of biological organization for which reaction norms were estimated and compared. Unless otherwise indicated, all LMMs were performed using the lmer function of the lme4 package in R (Bates et al. 2015), and the glht function of the multcomp package in R (Hothorn et al. 2008) was used for multiple pairwise comparisons using the false discovery rate (FDR) adjustment of *P*‐values. The Anova function of the car package in R was used to extract the Chisq and *P*‐values of each factor in the LMMs. We used R version 3.3.0 (R Core Team, 2017).

All leaf glucosinolates and floral volatile variables were ln(*x* + 1) transformed before analysis. Plasticity in leaf glucosinolates, floral morphology, and floral volatiles under noninfested and infested environments were evaluated following the next two approaches. (1) Comparisons within each selection treatment: These comparisons revealed how many phenotypic traits showed plasticity per selection treatment, and whether the effect was positive (increase upon infestation) or negative (decrease upon infestation). For this, we performed LMMs using each trait as the response variable in separate models, with the herbivore environment (two levels, “noninfested” and “infested”) and the interaction of herbivore environment × replicate as fixed factors and replicate as a random factor. (2) Comparisons between treatments: For these comparisons, we first calculated the difference between the values of noninfested and infested plants for each trait at the sibling plant‐pair level (the “sibling reaction norm”). We then performed LMMs using the sibling reaction norm of each trait as response variable, with selection treatment and the interaction of selection treatment × replicate as fixed factors and replicate as a random factor. We then performed multiple (Tukey HSD) pairwise comparisons with FDR adjustment of *P*‐values to figure out how selection treatments differed from each other. We also performed plasticity comparisons between treatments through a multivariate approach. For this, we used the sibling reaction norm values to perform LDAs using only the leaf glucosinolates (nine glucosinolates; *N* = 93), only the floral traits (19 traits; *N* = 78), and the leaf glucosinolates and floral traits combined (28 traits; *N* = 45). All values were ln(*x* + 1) transformed beforehand. This approach allowed us to reveal patterns of variation in the plasticity by selection treatment in multivariate space. Additionally, given our 2 × 2 factorial design, we also tested the influence of pollination (P), herbivory (H), and their interaction (P × H) as factors that could potentially explain the difference in the evolved levels of plasticity, both in multivariate or univariate approach. For the multivariate approach, we used the resulting discriminant functions from our above‐described LDAs. We then performed LMMs with each discriminant function as a response variable, and P, H, and P × H as fixed effects, and replicate as a random factor. For the univariate approach, we performed similar LMMs to test the impact of pollination (P), herbivory (H), and their interaction (P × H) but separately by plant trait (see Table S4). The LMMs used for testing the effect of P, H, and P × H above described were performed in the statistical package JMP (JMP^®^, Version 14, SAS Institute Inc.).

To test for an association between replicate mean trait values (from noninfested plants) and their mean reaction norm, we calculated the replicate mean reaction norm from the sibling reaction norm values, taking replicate as our population unit. We first calculated mean values for all traits (28 traits) per replicate for noninfested and infested plants. We then subtracted the mean infested from the mean noninfested values by replicate, obtaining so 12 “mean replicate reaction norm” values. The resulting dataset of mean replicate reaction norm values was obtained in absolute units to facilitate interpretation of the direction of the slope in the correlations. This dataset was merged with the dataset of the mean trait values for noninfested plants per replicate that also contained 12 mean values per trait. In this way, we were able to perform Pearson product‐moment correlations between replicate mean trait values (predictor) and replicate mean reaction norm values (response). We used the mean trait values of noninfested plants as the predictor variable, as these represent the constitutive values of a given trait that we used as a benchmark to compare its plastic response upon herbivory. For leaf glucosinolates, floral morphology traits, and floral volatiles, we used the ln(*x* + 1)‐transformed values to fulfill normality assumptions. However, because ln transformations can change the correlation pattern (Quinn and Keough 2002), we performed such correlations for all 28 traits with the nontransformed and the transformed data, and only those traits for which the correlation pattern and significance were held in both approaches were considered as a genuine correlation; only six traits fulfilled this criterion (Fig. 4). Pearson product‐moment correlations were estimated in R, and the plots were produced with the ggscatter function of the ggpubr package in R (version 3.3.0; R Development Core Team 2016).

Leaf volatiles of infested plants were analyzed via LDAs and LMMs. For the LDA, we used the 21 volatile compounds, and replicate was used to predefine groups to test the hypothesis that replicates within treatment should resemble each other more than across treatments as a result of similar selective pressures within each treatment (Ramos and Schiestl 2019). For univariate LMMs, we used each leaf volatile as a response variable and the fixed factors of treatment and treatment × replicate and replicate as the random factor. A significant treatment effect without an interaction effect would indicate consistent evolutionary changes across replicates. In contrast, a significant effect of the interaction of treatment × replicate would indicate inconsistent changes across replicates as a likely result of drift effects (Ramos and Schiestl 2019). LMMs for leaf volatiles were performed in JMP (JMP^®^, Version 14, SAS Institute Inc.). To test for parasitoid preferences for herbivore‐infested plants between treatments, we performed a negative binomial generalized linear mixed model using the glmmadmb function from the glmmADMB package in R (Bolker et al. 2009). The response variable was the number of wasps, treatment was included as a fixed factor, whereas plant replicate (A, B, and C), wasp replicate (12 replicates), and the interactions of treatment × plant replicate and treatment × wasp replicate were random factors.

## AUTHOR CONTRIBUTIONS

SER and FPS designed the study. SER performed the experiments. SER and FPS analyzed the data and wrote the manuscript.

## DATA ARCHIVING

Original data and R code has been archived in Dryad under https://doi.org/10.5061/dryad.59zw3r25n.

## CONFLICT OF INTEREST

The authors declare no conflict of interest.

2

Associate Editor: S. Wright

## Supporting information


**Figure S1**. Full‐sibling plants from each selection treatment at generation eight under two herbivore environments (noninfested and infested).
**Figure S2**. Evolutionary changes in the leaf volatiles of infested plants and parasitoid preferences.
**Table S1**. Summarized results of LMMs to evaluate within treatment herbivore‐induced plasticity for 28 plant traits.
**Table S2**. Summarized results of LMMs to evaluate differences between treatments in the plasticity of 28 plant traits using sibling reaction norm values.
**Table S3**. Results of the linear mixed models to test the effect of pollination, herbivory and the interaction of P*H on the sibling reaction norms.
**Table S4**. Results of the linear mixed models to test the effect of pollination, herbivory and the interaction of P*H on the sibling reaction norm.
**Table S5**. Univariate comparisons of 21 leaf volatiles from infested plants between treatments. Leaf volatiles (mean ± SD) were collected only from previously infested plants.
