# Supplementary material for: Herbivory and pollination impact on the evolution of herbivore‐induced plasticity in defense and floral traits
Source: Evol Lett. 2020 Oct 26;4(6):556–69. doi: 10.1002/evl3.200 (PMC7719550; doi:10.1002/evl3.200)
Supplement: Supplementary file 1 — Figure S1. Full‐sibling plants from each selection treatment at generation eight under two herbivore environments (noninfested and infested). Figure S2. Evolutionary changes in the leaf volatiles of infested plants and parasitoid preferences. Table S1. Summarized results of LMMs to evaluate within treatment herbivore‐induced plasticity for 28 plant traits. Table S2. Summarized results of LMMs to evaluate differences between treatments in the plasticity of 28 plant traits using sibling reaction norm values. Table S3. Results of the linear mixed models to test the effect of pollination, herbivory and the interaction of P*H on the sibling reaction norms. Table S4. Results of the linear mixed models to test the effect of pollination, herbivory and the interaction of P*H on the sibling reaction norm. Table S5. Univariate comparisons of 21 leaf volatiles from infested plants between treatments. Leaf volatiles (mean ± SD) were collected only from previously infested plants. [file EVL3-4-556-s001.docx]

*Supplementary Information*

**Herbivory and pollination impact on the evolution of herbivore-induced plasticity in defense- and floral traits**

Sergio E. Ramos^1,2^, Florian P. Schiestl^1,3^

**Affiliations:**

^1^Department of Systematic and Evolutionary Botany, University of Zurich, Zollikerstrasse 107, CH-8008 Zurich, Switzerland.

^2^Current address: Laboratory of Plant Evolution & Ecology, Department of Biological Sciences, University of Pittsburgh, PA, USA.

^3^ Corresponding author

florian.schiestl@systbot.uzh.ch

s.ramos.castro@gmail.com

**Contents:**

**Supplementary text:**

Quantification of leaf glucosinolates

Quantification of floral and leaf volatiles

**Supplementary results.**

Leaf volatiles of infested plants and parasitoid preferences

**Figure S1, S2**

**Tables S1 to S5**

**Supplementary text**

**Quantification of leaf glucosinolates**. For glucosinolate quantification, around 100 mg of fresh leaf tissue per plant was immediately frozen in liquid nitrogen to avoid myrosinase activation. Samples were weighed (ca. 100 mg) and ground to a fine powder with a TissueLyser II (Qiagen NV, Düsseldorf, Germany) using 3 glass balls. The samples were grinded for 35 seconds and cooled down again in liquid nitrogen for ca. 20 seconds before grinding them for another 35 seconds at a frequency of 30 oscillations per second. 1 ml of a solution of sinalbin (5 μg ml^─1^; internal standard) and ice cold MeOH:water (70:30; Methanol gradient grade 235 nm) was added to the frozen leaf powder. Samples were shaked with a vortex for 5 s and immediately incubated at 85°C for 10 min in a block heater and simultaneously shaked at 600 rpm (Eppendorf Thermomixer™ comfort). For further extraction, samples were put in an ultrasonic bath for 10 min (Advantage‐Lab, Typ AL 04‐04). Extracts were then centrifuged at 14000 rpm for 10 min (Sorvall RMC 14, USA) and the supernatant was transferred to a new tube and stored at ‐20 °C until UHPLC analysis. UHPLC/MS analysis for identification and quantification of glucosinolates from the samples was done as described in Schiestl et al. (2014). Nine glucosinolates were identified in our leaf samples and all were considered for statistical analyses. Total sample size was *N* = 210 samples.

**Quantification of floral and leaf volatiles**. Quantification of flower and leaf volatiles was conducted by using gas chromatography with mass selective detection (GC–MSD; Agilent 6890N; Agilent Technologies, Palo Alto, USA). Injection was done with a Gerstel thermal desorption unit (TDU3, Gerstel) and a cold injection system (CIS; Gerstel). For thermodesorption, the TDU was heated from 30 to 240 °C at a rate of 60 °C min^─1^ and held at a final temperature for 1 min. The CIS was set to ─ 150 °C during the trapping of eluting compounds from the TDU. For injection, the CIS was heated to 250 °C at a rate of 12 °Cs^─1^, and the final temperature was held for 3 min. The GC was equipped with a HP-5 column (0.25 mm diameter, 0.25 mm film thickness, 15 m length), and helium was used as carrier gas at a flow rate of 2 ml min^─1^. Compound identification and quantification were done with the Agilent MSD chemstation software (v.E.01.00, Agilent Technologies AG, Santa Clara, USA) and by comparing the mass spectra of the samples with those of the National Institute of Standards and Technology (NIST). Quantification of compounds was obtained through measurement of peak areas of selected target ions specific to the individual scent compounds; peak areas were converted into absolute amounts using calibration curves previously obtained for each compound using synthetic compounds in three different concentrations. For data analysis we only included volatiles that were present in significantly higher amounts (*P*<0.05) than in the respective air control. All volatiles were standardized in units of pg^-1^ flower^-1^ L sampled air. The final matrix included 13 flower volatiles and a total sample size of *N* = 173 samples, and 21 leaf volatiles and a total sample size of *N* = 174 samples

**Supplementary results.**

*Leaf volatiles of infested plants and parasitoid preferences*

Upon herbivory, plants increase their emission of volatiles (VOCs) in their leaves, which is used by predators or parasitoids as a signal for the presence of prey (Heil 2010; Dicke 2016). Multivariate analyses with the leaf volatiles of infested plants *via* linear discriminant analyses using replicate as grouping factor, showed that leaf volatiles were more similar among replicates across treatments than within treatment. This result suggest that induction of leaf volatiles did not evolved in a divergent fashion during the previous evolutionary experiment (Fig. S2A). The similarity of replicates across different treatment groups likely mirrors genetic relationship, as the same full sib families were used within replicates at the onset of the experiment (Ramos and Schiestl 2019). From univariate analyses per volatile, we found that only 1 out of 21 herbivore-induced leaf volatiles was different between treatments; this volatile, linalool, showed a higher herbivore-induced emission in the plants of hand pollination without herbivory (H_H_–) (Fig. S2B). From the six-choice bioassays we found that wasps preferred two groups of infested plants over empty cylinders, namely plants with bee pollination and no herbivory (B_H_– vs empty glass: GLMM negative binomial, z-value = -2.55, *P* = 0.011), and plants of hand pollination without herbivory (H_H_– vs empty glass: GLMM negative binomial, z-value = -1.99, *P* = 0.046) (Fig. S2C). Furthermore, preferences of parasitoid wasps were not different between selection treatments, suggesting that indirect defenses did not change in the course of the experimental evolution. The likely explanation is that parasitoid *Cotesia* wasps were not used during our previous experimental evolution study, and thus there was no selection on plants and their induced leaf volatiles to adapt to the wasp’s preferences. Our results show that evolutionary changes in the plasticity of the direct defenses (i.e. leaf glucosinolates) do not necessarily affect indirect defenses, perhaps because of a lack of physiological connections between them.

**
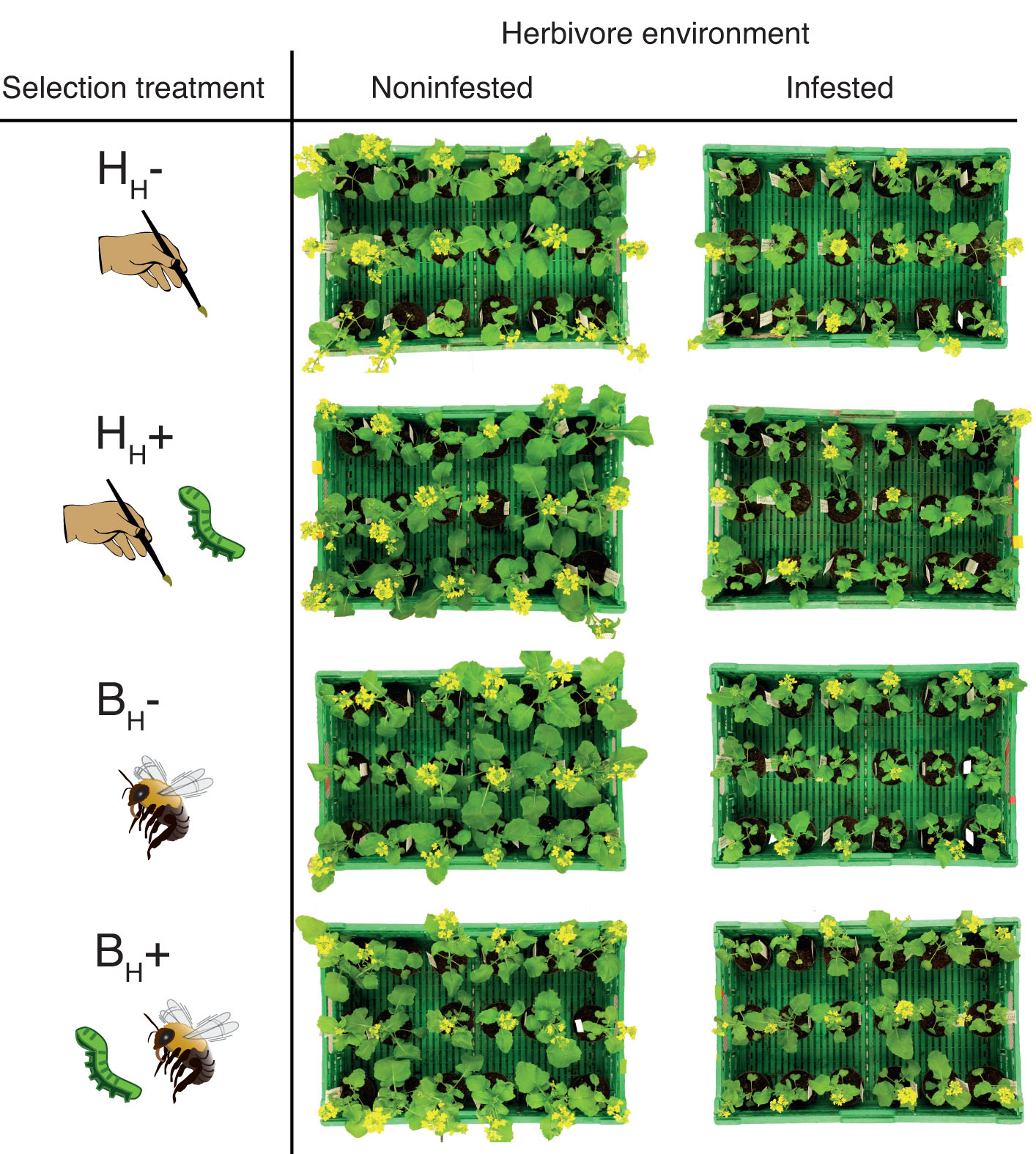
**

**Figure S1**. Full-sibling plants from each selection treatment at generation eight under two herbivore environments (noninfested and infested). The pictures show 20-day old *Brassica rapa* plants from above to allow a better appreciation of the effects of pre-flowering *Pieris brassicae* larvae herbivory applied during 72 h on infested plants. Larvae were removed from the infested plants three days before taking these pictures, thus their relatively short plant size compared to noninfested plants is the result of herbivore-induced metabolic changes rather than tissue removal. The plants in the pictures do not represent the total number of plants used per treatment.

**
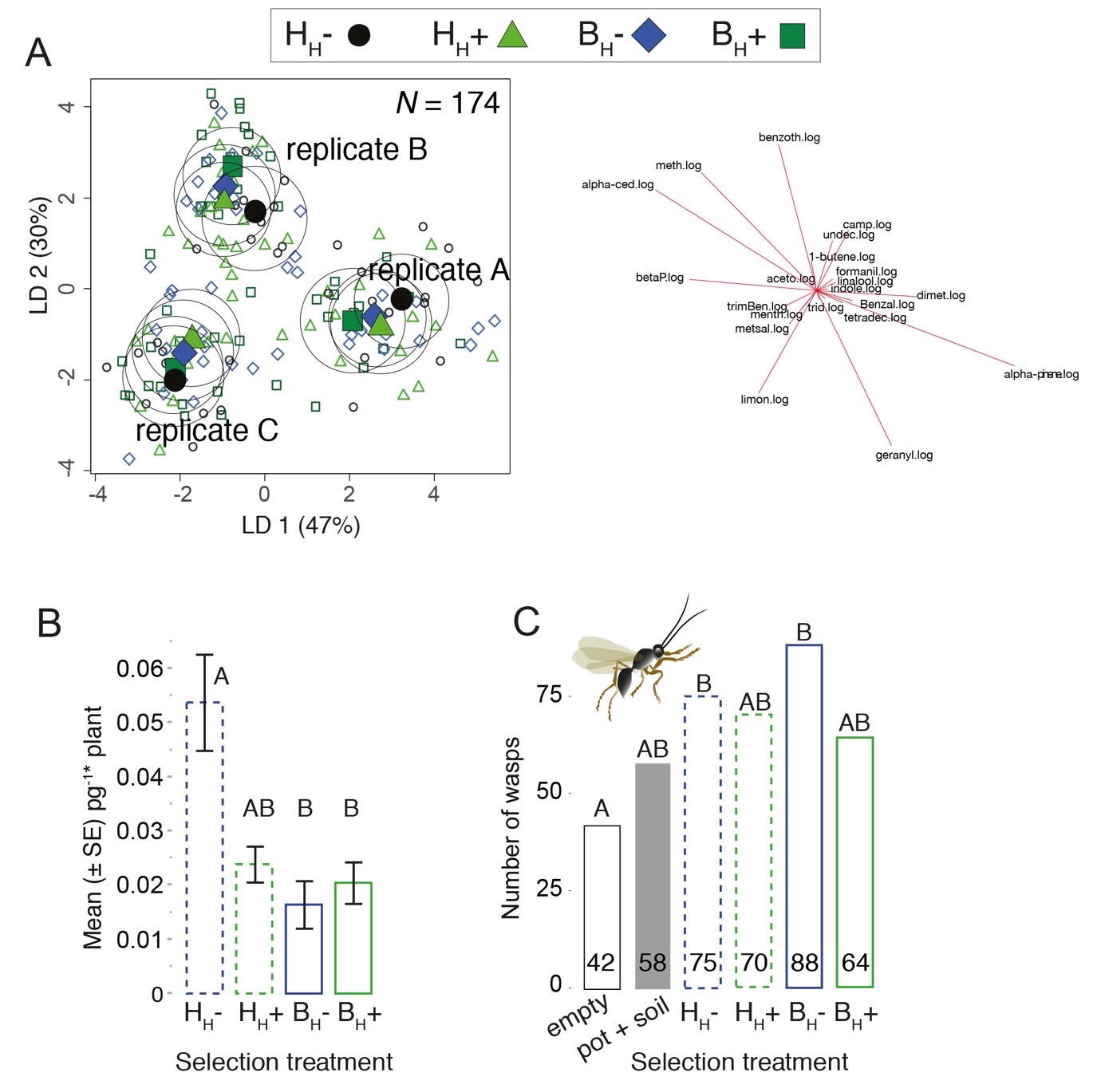
**

**Figure S2**. Evolutionary changes in the leaf volatiles of infested plants and parasitoid preferences. (A) Multivariate comparisons of leaf volatiles from infested plants. The linear discriminant analysis was predefined by replicate, and 21 leaf volatiles were used (see list of volatiles in Table S5). Enlarged filled symbols indicate replicate centroids. The LDA shows that replicates between treatments resemble each other more than replicates within treatments (Wilks' λ for replicate = 0.953, df = 11, *F* = 8.074, *P* = 0.216). Sample size (*N*) is shown on the upper-right corner. (B) Linalool emission in the leaves of infested plants was higher in hand pollinated plants without herbivory (H_H_–) compared to plants of bee pollination without and with herbivory (B_H_+ and B_H_– respectively). Different letters indicate group differences after Tukey HSD *post-hoc* test (See Table S5). (C) *Cotesia glomerata* wasps did not show specific preferences for herbivore-infested plants with different treatment history. Numbers in the bars indicate the absolute number of wasps per treatment. Different letters indicate group differences (GLMM, AIC = 1064.3, *N* = 397 wasps)

**Table S1**. Summarized results of LMMs to evaluate within treatment herbivore-induced plasticity for 28 plant traits. Analyses of each variable were performed separately by selection treatment with plants of generation eight. Sources of variation are herbivory environment, ‘H’ (‘noninfested’ and ‘infested’), replicate, ‘R’ (random factor), and the interaction of herbivory*replicate, ‘H*R’. *N* = sample size per trait. Treatments: H_H_– = hand-pollination and no herbivory; H_H_+, hand-pollination and constant herbivory; B_H_–, bee-pollination and no herbivory; B_H_+, bee-pollination and constant herbivory.

|  |  | H_H_– | | | H_H+_ | | | B_H_– | | | B_H+_ | | |  |
| --- | --- | --- | --- | --- | --- | --- | --- | --- | --- | --- | --- | --- | --- | --- |
| Trait | Source | df | Chisq | *P* | df | Chisq | *P* | df | Chisq | *P* | df | Chisq | *P* | |
| **Leaf glucosinolates** |  | *N =* 52 | | | *N* = 49 | | | *N* = 55 | | | *N* = 54 | | |  |
| Glucobrassicanapin | H | 1 | 19.037 | <.0001 | 1 | 0.025 | 0.8739 | 1 | 12.984 | <0.001 | 1 | 21.459 | <.0001 | |
|  | R | 2 | 6.272 | 0.0435 | 2 | 9.008 | 0.0111 | 2 | 3.579 | 0.1670 | 2 | 3.597 | 0.1656 | |
|  | H*R | 2 | 12.779 | 0.0017 | 2 | 11.936 | 0.0026 | 2 | 8.143 | 0.0170 | 2 | 1.129 | 0.5688 | |
| Glucoerucin | H | 1 | 0.869 | 0.3513 | 1 | 0.004 | 0.9506 | 1 | 4.096 | 0.0430 | 1 | 0.597 | 0.4399 | |
|  | R | 2 | 2.373 | 0.3052 | 2 | 1.999 | 0.3680 | 2 | 3.112 | 0.2110 | 2 | 1.885 | 0.3896 | |
|  | H*R | 2 | 3.184 | 0.2035 | 2 | 0.300 | 0.8606 | 2 | 6.522 | 0.0384 | 2 | 0.411 | 0.8143 | |
| Gluconapin | H | 1 | 22.419 | <.0001 | 1 | 0.616 | 0.4326 | 1 | 13.013 | <0.001 | 1 | 12.157 | <0.001 | |
|  | R | 2 | 1.238 | 0.5385 | 2 | 1.330 | 0.5143 | 2 | 0.009 | 0.9957 | 2 | 2.597 | 0.2730 | |
|  | H*R | 2 | 12.764 | 0.0017 | 2 | 3.398 | 0.1828 | 2 | 4.719 | 0.0945 | 2 | 0.215 | 0.8982 | |
| Gluconasturtiin | H | 1 | 0.541 | 0.4622 | 1 | 1.867 | 0.1718 | 1 | 0.485 | 0.4863 | 1 | 0.021 | 0.8856 | |
|  | R | 2 | 1.675 | 0.4328 | 2 | 0.889 | 0.6410 | 2 | 0.946 | 0.6232 | 2 | 0.466 | 0.7922 | |
|  | H*R | 2 | 1.891 | 0.3886 | 2 | 5.415 | 0.0667 | 2 | 3.469 | 0.1765 | 2 | 1.315 | 0.5182 | |
| Glucoraphanin | H | 1 | 3.060 | 0.0802 | 1 | 0.153 | 0.6958 | 1 | 4.953 | 0.0260 | 1 | 0.100 | 0.7524 | |
|  | R | 2 | 1.582 | 0.4534 | 2 | 1.051 | 0.5913 | 2 | 3.704 | 0.1569 | 2 | 1.456 | 0.4828 | |
|  | H*R | 2 | 6.713 | 0.0349 | 2 | 0.430 | 0.8065 | 2 | 3.150 | 0.2071 | 2 | 0.314 | 0.8548 | |
| Glucobrassicin | H | 1 | 3.280 | 0.0701 | 1 | 0.777 | 0.3780 | 1 | 0.158 | 0.6909 | 1 | 0.536 | 0.4640 | |
|  | R | 2 | 1.235 | 0.5394 | 2 | 4.010 | 0.1346 | 2 | 1.858 | 0.3950 | 2 | 5.356 | 0.0687 | |
|  | H*R | 2 | 1.277 | 0.5281 | 2 | 1.712 | 0.4248 | 2 | 1.472 | 0.4790 | 2 | 5.860 | 0.0534 | |
| Hydroxyglucobrassicin | H | 1 | 2.469 | 0.1161 | 1 | 3.248 | 0.0715 | 1 | 0.262 | 0.6091 | 1 | 1.526 | 0.2168 | |
|  | R | 2 | 1.329 | 0.5144 | 2 | 6.004 | 0.0497 | 2 | 4.208 | 0.1220 | 2 | 5.637 | 0.0597 | |
|  | H*R | 2 | 0.438 | 0.8033 | 2 | 4.880 | 0.0872 | 2 | 1.967 | 0.3741 | 2 | 8.282 | 0.0159 | |
| Methoxyglucobrassicin | H | 1 | 0.194 | 0.6601 | 1 | 0.002 | 0.9690 | 1 | 0.217 | 0.6415 | 1 | 0.092 | 0.7622 | |
|  | R | 2 | 3.091 | 0.2132 | 2 | 0.840 | 0.6571 | 2 | 0.900 | 0.6375 | 2 | 0.810 | 0.6670 | |
|  | H*R | 2 | 0.844 | 0.6557 | 2 | 0.046 | 0.9774 | 2 | 1.041 | 0.5944 | 2 | 1.906 | 0.3856 | |
| Neoglucobrassicin | H | 1 | 34.969 | <.0001 | 1 | 26.015 | <.0001 | 1 | 16.557 | <.0001 | 1 | 37.892 | <.0001 | |
|  | R | 2 | 12.155 | 0.0023 | 2 | 4.391 | 0.1113 | 2 | 1.391 | 0.4989 | 2 | 13.061 | 0.0015 | |
|  | H*R | 2 | 9.016 | 0.0110 | 2 | 2.864 | 0.2388 | 2 | 0.613 | 0.7359 | 2 | 11.698 | 0.0029 | |
| **Flower morphometry** |  | *N* = 72 | | | *N* = 67 | | | *N* = 74 | | | *N* = 86 | | |  |
| Sepal length | H | 1 | 1.627 | 0.2022 | 1 | 19.841 | <.0001 | 1 | 12.651 | <0.001 | 1 | 19.767 | <.0001 | |
|  | R | 2 | 2.026 | 0.3631 | 2 | 1.360 | 0.5067 | 2 | 4.254 | 0.1192 | 2 | 2.694 | 0.2600 | |
|  | H*R | 2 | 0.835 | 0.6587 | 2 | 9.913 | 0.0070 | 2 | 3.033 | 0.2195 | 2 | 13.330 | 0.0013 | |
| Petal length | H | 1 | 5.289 | 0.0215 | 1 | 10.143 | 0.0014 | 1 | 10.016 | 0.0016 | 1 | 13.214 | <0.001 | |
|  | R | 2 | 3.244 | 0.1975 | 2 | 0.843 | 0.6562 | 2 | 3.843 | 0.1464 | 2 | 3.942 | 0.1393 | |
|  | H*R | 2 | 2.243 | 0.3258 | 2 | 2.083 | 0.3529 | 2 | 0.770 | 0.6806 | 2 | 19.639 | <0.001 | |
| Petal width | H | 1 | 6.757 | 0.0093 | 1 | 22.390 | <.0001 | 1 | 17.320 | <.0001 | 1 | 18.590 | <.0001 | |
|  | R | 2 | 1.171 | 0.5569 | 2 | 4.238 | 0.1201 | 2 | 6.388 | 0.0410 | 2 | 10.689 | 0.0048 | |
|  | H*R | 2 | 4.430 | 0.1091 | 2 | 13.338 | 0.0013 | 2 | 5.220 | 0.0735 | 2 | 21.413 | <.0001 | |
| Pistil length | H | 1 | 1.803 | 0.1793 | 1 | 5.743 | 0.0166 | 1 | 2.488 | 0.1147 | 1 | 2.068 | 0.1505 | |
|  | R | 2 | 1.792 | 0.4082 | 2 | 0.900 | 0.6375 | 2 | 4.818 | 0.0899 | 2 | 3.442 | 0.1789 | |
|  | H*R | 2 | 2.342 | 0.3100 | 2 | 5.315 | 0.0701 | 2 | 2.191 | 0.3345 | 2 | 5.976 | 0.0504 | |
| Long stamen | H | 1 | 3.925 | 0.0476 | 1 | 3.448 | 0.0633 | 1 | 9.148 | 0.0025 | 1 | 6.652 | 0.0099 | |
|  | R | 2 | 2.936 | 0.2304 | 2 | 3.341 | 0.1882 | 2 | 4.429 | 0.1092 | 2 | 5.878 | 0.0529 | |
|  | H*R | 2 | 1.505 | 0.4713 | 2 | 3.802 | 0.1495 | 2 | 4.680 | 0.0963 | 2 | 14.294 | <0.001 | |
| Short stamen | H | 1 | 2.021 | 0.1552 | 1 | 0.071 | 0.7907 | 1 | 4.947 | 0.0261 | 1 | 8.560 | 0.0034 | |
|  | R | 2 | 2.543 | 0.2805 | 2 | 1.755 | 0.4158 | 2 | 1.963 | 0.3748 | 2 | 3.332 | 0.1890 | |
|  | H*R | 2 | 0.049 | 0.9759 | 2 | 1.025 | 0.5991 | 2 | 2.220 | 0.3296 | 2 | 9.759 | 0.0076 | |
| **Flower volatiles** |  | *N* = 40 | | | *N* = 40 | | | *N* = 42 | | | *N* = 51 | | |  |
| Benzaldehyde | H | 1 | 0.001 | 0.9769 | 1 | 0.025 | 0.8748 | 1 | 0.213 | 0.6446 | 1 | 0.385 | 0.5352 | |
|  | R | 2 | 1.576 | 0.4548 | 2 | 2.076 | 0.3542 | 2 | 0.306 | 0.8583 | 2 | 1.152 | 0.5622 | |
|  | H*R | 2 | 0.515 | 0.7729 | 2 | 0.125 | 0.9394 | 2 | 0.356 | 0.8368 | 2 | 0.227 | 0.8929 | |
| 1-Butene-4-Iisothyocyanate | H | 1 | 1.027 | 0.3110 | 1 | 0.688 | 0.4069 | 1 | 1.375 | 0.2410 | 1 | 1.135 | 0.2868 | |
|  | R | 2 | 1.003 | 0.6058 | 2 | 1.458 | 0.4825 | 2 | 2.756 | 0.2521 | 2 | 6.428 | 0.0402 | |
|  | H*R | 2 | 0.044 | 0.9784 | 2 | 0.272 | 0.8727 | 2 | 4.777 | 0.0918 | 2 | 9.253 | 0.0098 | |
| 6-Methyl-5-hepten-2-one | H | 1 | 1.062 | 0.3028 | 1 | 3.001 | 0.0832 | 1 | 0.245 | 0.6210 | 1 | 0.124 | 0.7249 | |
|  | R | 2 | 4.510 | 0.1049 | 2 | 0.372 | 0.8304 | 2 | 2.043 | 0.3600 | 2 | 1.467 | 0.4802 | |
|  | H*R | 2 | 4.049 | 0.1320 | 2 | 2.241 | 0.3261 | 2 | 0.672 | 0.7147 | 2 | 0.025 | 0.9878 | |
| *(Z)*-3-Hexenyl acetate | H | 1 | 0.074 | 0.7863 | 1 | 0.849 | 0.3569 | 1 | 6.182 | 0.0129 | 1 | 0.181 | 0.6708 | |
|  | R | 2 | 1.880 | 0.3907 | 2 | 0.724 | 0.6964 | 2 | 2.370 | 0.3057 | 2 | 3.372 | 0.1853 | |
|  | H*R | 2 | 1.958 | 0.3757 | 2 | 2.956 | 0.2281 | 2 | 3.240 | 0.1979 | 2 | 1.094 | 0.5787 | |
| Phenylacetaldehyde | H | 1 | 0.011 | 0.9168 | 1 | 2.428 | 0.1192 | 1 | 5.197 | 0.0226 | 1 | 13.973 | <0.001 | |
|  | R | 2 | 0.914 | 0.6331 | 2 | 0.662 | 0.7181 | 2 | 1.298 | 0.5225 | 2 | 0.291 | 0.8644 | |
|  | H*R | 2 | 2.520 | 0.2836 | 2 | 2.814 | 0.2449 | 2 | 0.957 | 0.6196 | 2 | 10.063 | 0.0065 | |
| Methyl benzoate | H | 1 | 2.000 | 0.1573 | 1 | 0.179 | 0.6720 | 1 | 3.041 | 0.0812 | 1 | 1.369 | 0.2420 | |
|  | R | 2 | 1.302 | 0.5215 | 2 | 2.079 | 0.3537 | 2 | 1.531 | 0.4651 | 2 | 0.120 | 0.9419 | |
|  | H*R | 2 | 1.602 | 0.4489 | 2 | 2.930 | 0.2311 | 2 | 1.037 | 0.5955 | 2 | 5.139 | 0.0766 | |
| Benzyl nitrile | H | 1 | 0.184 | 0.6676 | 1 | 0.063 | 0.8012 | 1 | 6.668 | 0.0098 | 1 | 1.843 | 0.1746 | |
|  | R | 2 | 0.865 | 0.6489 | 2 | 0.925 | 0.6297 | 2 | 1.477 | 0.4779 | 2 | 0.276 | 0.8713 | |
|  | H*R | 2 | 2.281 | 0.3197 | 2 | 0.267 | 0.8752 | 2 | 2.117 | 0.3470 | 2 | 3.521 | 0.1720 | |
| Methyl salicylate | H | 1 | 0.687 | 0.4074 | 1 | 0.912 | 0.3396 | 1 | 3.554 | 0.0594 | 1 | 0.024 | 0.8774 | |
|  | R | 2 | 2.305 | 0.3159 | 2 | 3.309 | 0.1912 | 2 | 2.598 | 0.2728 | 2 | 0.123 | 0.9406 | |
|  | H*R | 2 | 0.337 | 0.8449 | 2 | 1.669 | 0.4342 | 2 | 3.294 | 0.1927 | 2 | 1.939 | 0.3794 | |
| 2-Aminobenzaldehyde | H | 1 | 0.277 | 0.5990 | 1 | 7.835 | 0.0051 | 1 | 2.042 | 0.1531 | 1 | 0.715 | 0.3979 | |
|  | R | 2 | 2.156 | 0.3404 | 2 | 5.071 | 0.0792 | 2 | 1.337 | 0.5124 | 2 | 1.528 | 0.4657 | |
|  | H*R | 2 | 1.539 | 0.4633 | 2 | 7.320 | 0.0257 | 2 | 0.707 | 0.7023 | 2 | 0.453 | 0.7972 | |
| *p*-Anisaldehyde | H | 1 | 0.042 | 0.8375 | 1 | 0.523 | 0.4696 | 1 | 1.014 | 0.3140 | 1 | 0.001 | 0.9781 | |
|  | R | 2 | 0.293 | 0.8638 | 2 | 0.356 | 0.8370 | 2 | 0.549 | 0.7601 | 2 | 1.390 | 0.4990 | |
|  | H*R | 2 | 2.526 | 0.2828 | 2 | 1.348 | 0.5096 | 2 | 0.638 | 0.7268 | 2 | 0.732 | 0.6934 | |
| Indole | H | 1 | 0.001 | 0.9709 | 1 | 2.965 | 0.0851 | 1 | 7.410 | 0.0065 | 1 | 0.850 | 0.3566 | |
|  | R | 2 | 0.653 | 0.7213 | 2 | 1.245 | 0.5367 | 2 | 1.710 | 0.4252 | 2 | 1.648 | 0.4388 | |
|  | H*R | 2 | 0.965 | 0.6174 | 2 | 2.634 | 0.2680 | 2 | 2.684 | 0.2613 | 2 | 0.288 | 0.8660 | |
| Methyl anthranilate | H | 1 | 0.000 | 0.9897 | 1 | 0.011 | 0.9148 | 1 | 2.218 | 0.1364 | 1 | 0.003 | 0.9581 | |
|  | R | 2 | 1.162 | 0.5593 | 2 | 1.006 | 0.6047 | 2 | 0.998 | 0.6072 | 2 | 2.409 | 0.2999 | |
|  | H*R | 2 | 0.499 | 0.7794 | 2 | 0.180 | 0.9138 | 2 | 1.138 | 0.5661 | 2 | 1.576 | 0.4547 | |
| (*E,E*)-α-Farnesene | H | 1 | 0.720 | 0.3961 | 1 | 0.302 | 0.5827 | 1 | 5.302 | 0.0213 | 1 | 0.315 | 0.5745 | |
|  | R | 2 | 2.125 | 0.3456 | 2 | 1.641 | 0.4402 | 2 | 2.047 | 0.3594 | 2 | 2.491 | 0.2878 | |
|  | H*R | 2 | 0.347 | 0.8408 | 2 | 1.300 | 0.5221 | 2 | 2.396 | 0.3018 | 2 | 0.277 | 0.8707 | |

**Table S2**. Summarized results of LMMs to evaluate differences between treatments in the plasticity of 28 plant traits using sibling reaction norm values. The mean ± s.d. per treatment is the result of the mean noninfested minus mean infested values in the following units: glucosinolates are in $\mu$g ml^-1^100 mg leaf tissue, morphometry in mm and floral VOCs in pg^-1^ flower^-1^ L. Sources of variation are selection treatment ‘T’, and the interaction of selection treatment*replicate ‘T*R’ as fixed factors, with replicate ‘R’ as random factor. Values in bold indicate a significant effect of ‘T’. Different letters indicate the results of HSD Tukey *post-hoc* tests. Treatments: H_H_– = hand-pollination and no herbivory; H_H_+, hand-pollination and constant herbivory; B_H_–, bee-pollination and no herbivory; B_H_+, bee-pollination and constant herbivory.

| Trait | *N* | H_H_– | | H_H+_ | | B_H_– | | B_H+_ | | Source | | df | | Chisq | | *P* | |  |
| --- | --- | --- | --- | --- | --- | --- | --- | --- | --- | --- | --- | --- | --- | --- | --- | --- | --- | --- |
| **Leaf glucosinolates** |  |  | |  | |  | |  | |  | |  | |  | |  | |  |
| Glucobrassicanapin | 93 | **-60.98 ± 89.87** | | **-28.16 ± 104.02** | | **-126.05 ± 146.8** | | **-119.97 ± 125.3** | | **T** | | **3** | | **15.586** | | **0.001** | |  |
|  |  | A | | B | | A | | A | | R | | 2 | | 4.972 | | 0.133 | |  |
|  |  |  | |  | |  | |  | | T*R | | 6 | | 22.381 | | 0.001 | |  |
| Glucoerucin | 93 | 0.47 ± 2.1 | | 1.04 ± 5.04 | | -0.04 ± 0.39 | | -0.05 ± 0.26 | | T | | 3 | | 0.571 | | 0.903 | |  |
|  |  |  | |  | |  | |  | | R | | 2 | | 3.838 | | 0.147 | |  |
|  |  |  | |  | |  | |  | | T*R | | 6 | | 8.288 | | 0.218 | |  |
| Gluconapin | 93 | **-1622.47 ± 2038.18** | | **-729.72 ± 2516.33** | | **-2016.17 ± 2150.47** | | **-1860.5 ± 1944.8** | | **T** | | **3** | | **9.666** | | **0.022** | |  |
|  |  | A | | B | | AB | | A | | R | | 2 | | 3.396 | | 0.123 | |  |
|  |  |  | |  | |  | |  | | T*R | | 6 | | 14.297 | | 0.026 | |  |
| Gluconasturtiin | 93 | 18.46 ± 74.57 | | 23.35 ± 70.15 | | -18.14 ± 58.17 | | 2.68 ± 52.19 | | T | | 3 | | 2.864 | | 0.413 | |  |
|  |  |  | |  | |  | |  | | R | | 2 | | 2.076 | | 0.354 | |  |
|  |  |  | |  | |  | |  | | T*R | | 6 | | 11.062 | | 0.086 | |  |
| Glucoraphanin | 93 | 0.11 ± 8.93 | | -1.86 ± 7.9 | | -2.99 ± 6.82 | | -1.41 ± 7.5 | | T | | 3 | | 1.064 | | 0.786 | |  |
|  |  |  | |  | |  | |  | | R | | 2 | | 0.526 | | 0.769 | |  |
|  |  |  | |  | |  | |  | | T*R | | 6 | | 7.811 | | 0.252 | |  |
| Glucobrassicin | 93 | 12.03 ± 21.89 | | -5.84 ± 39.07 | | -3.71 ± 33.74 | | -3.81 ± 31.56 | | T | | 3 | | 6.567 | | 0.087 | |  |
|  |  |  | |  | |  | |  | | R | | 2 | | 1.841 | | 0.398 | |  |
|  |  |  | |  | |  | |  | | T*R | | 6 | | 12.622 | | 0.049 | |  |
| Hydroxyglucobrassicin | 93 | **0.88 ± 1.58** | | **-0.8 ± 3.07** | | **-0.29 ± 2.2** | | **-0.18 ± 2** | | **T** | | **3** | | **12.442** | | **0.006** | |  |
|  |  | A | | B | | AB | | AB | | R | | 2 | | 0.442 | | 0.801 | |  |
|  |  |  | |  | |  | |  | | T*R | | 6 | | 17.417 | | 0.019 | |  |
| Methoxyglucobrassicin | 93 | 4.76 ± 6.51 | | 2.88 ± 31.76 | | 0.69 ± 19.29 | | 3.52 ± 15.55 | | T | | 3 | | 0.174 | | 0.982 | |  |
|  |  |  | |  | |  | |  | | R | | 2 | | 4.386 | | 0.112 | |  |
|  |  |  | |  | |  | |  | | T*R | | 6 | | 11.752 | | 0.082 | |  |
| Neoglucobrassicin | 93 | -28.42 ± 39.26 | | -30.46 ± 33.27 | | -43.46 ± 109.03 | | -43.6 ± 109.48 | | T | | 3 | | 1.502 | | 0.682 | |  |
|  |  |  | |  | |  | |  | | R | | 2 | | 0.500 | | 0.779 | |  |
|  |  |  | |  | |  | |  | | T*R | | 6 | | 7.871 | | 0.248 | |  |
| **Flower morphometry** | | |  | |  | |  | |  | |  | |  | |  | |  | |
| Sepal length | 149 | 0.18 ± 0.54 | | 0.33 ± 0.48 | | 0.39 ± 0.54 | | 0.35 ± 0.59 | | T | | 3 | | 5.783 | | 0.123 | |  |
|  |  |  | |  | |  | |  | | R | | 2 | | 1.306 | | 0.520 | |  |
|  |  |  | |  | |  | |  | | T*R | | 6 | | 5.743 | | 0.453 | |  |
| Petal length | 149 | 0.34 ± 0.6 | | 0.44 ± 0.59 | | 0.61 ± 0.55 | | 0.21 ± 0.73 | | T | | 3 | | 0.352 | | 0.950 | |  |
|  |  |  | |  | |  | |  | | R | | 2 | | 0.368 | | 0.832 | |  |
|  |  |  | |  | |  | |  | | T*R | | 6 | | 12.055 | | 0.061 | |  |
| Petal width | 149 | 0.31 ± 0.58 | | 0.45 ± 0.62 | | 0.6 ± 0.65 | | 0.35 ± 0.84 | | T | | 3 | | 3.294 | | 0.349 | |  |
|  |  |  | |  | |  | |  | | R | | 2 | | 1.536 | | 0.464 | |  |
|  |  |  | |  | |  | |  | | T*R | | 6 | | 5.959 | | 0.428 | |  |
| Pistil length | 149 | 0.38 ± 1 | | 0.48 ± 0.83 | | 0.54 ± 0.79 | | 0.05 ± 1.06 | | T | | 3 | | 0.339 | | 0.953 | |  |
|  |  |  | |  | |  | |  | | R | | 2 | | 1.178 | | 0.555 | |  |
|  |  |  | |  | |  | |  | | T*R | | 6 | | 2.900 | | 0.821 | |  |
| Large stamen | 149 | 0.19 ± 0.57 | | 0.27 ± 0.5 | | 0.24 ± 0.55 | | 0.02 ± 0.75 | | T | | 3 | | 0.898 | | 0.826 | |  |
|  |  |  | |  | |  | |  | | R | | 2 | | 2.053 | | 0.358 | |  |
|  |  |  | |  | |  | |  | | T*R | | 6 | | 10.666 | | 0.099 | |  |
| Short stamen | 149 | 0.28 ± 0.54 | | 0.18 ± 0.77 | | 0.23 ± 0.61 | | 0.19 ± 0.76 | | T | | 3 | | 3.963 | | 0.265 | |  |
|  |  |  | |  | |  | |  | | R | | 2 | | 1.655 | | 0.437 | |  |
|  |  |  | |  | |  | |  | | T*R | | 6 | | 9.153 | | 0.165 | |  |
| **Flower volatiles** |  |  | |  | |  | |  | |  | |  | |  | |  | |  |
| Benzaldehyde | 84 | -119.3 ± 362.86 | | -51.28 ± 389.4 | | -15.48 ± 473.67 | | -29.05 ± 190.86 | | T | | 3 | | 0.575 | | 0.902 | |  |
|  |  |  | |  | |  | |  | | R | | 2 | | 0.415 | | 0.813 | |  |
|  |  |  | |  | |  | |  | | T*R | | 6 | | 2.225 | | 0.898 | |  |
| 1-Butene-4-isothyocyanate | 84 | -26.22 ± 62 | | 18.08 ± 127.64 | | -30.42 ± 71.02 | | 20.47 ± 85.09 | | T | | 3 | | 4.977 | | 0.174 | |  |
|  |  |  | |  | |  | |  | | R | | 2 | | 2.020 | | 0.364 | |  |
|  |  |  | |  | |  | |  | | T*R | | 6 | | 9.475 | | 0.149 | |  |
| 6-Methyl-5-hepten-2-one | 84 | -14.47 ± 46.32 | | -0.02 ± 44.48 | | 2.43 ± 51.07 | | -7.9 ± 42.98 | | T | | 3 | | 6.193 | | 0.103 | |  |
|  |  |  | |  | |  | |  | | R | | 2 | | 0.577 | | 0.749 | |  |
|  |  |  | |  | |  | |  | | T*R | | 6 | | 8.189 | | 0.225 | |  |
| *(Z)*-3-Hexenyl acetate | 84 | **-7.08 ± 32.16** | | **2.07 ± 37.63** | | **19.28 ± 40.03** | | **-11.25 ± 35.92** | | **T** | | **3** | | **8.267** | | **0.046** | |  |
|  |  | AB | | A | | B | | AB | | R | | 2 | | 3.381 | | 0.184 | |  |
|  |  |  | |  | |  | |  | | T*R | | 6 | | 9.506 | | 0.147 | |  |
| Phenylacetaldehyde | 84 | **30.27 ± 137.23** | | **40.88 ± 180.78** | | **84.87 ± 175.28** | | **62.41 ± 198.73** | | **T** | | **3** | | **8.451** | | **0.038** | |  |
|  |  | A | | AB | | AB | | B | | R | | 2 | | 0.741 | | 0.690 | |  |
|  |  |  | |  | |  | |  | | T*R | | 6 | | 7.116 | | 0.310 | |  |
| Methyl benzoate | 84 | 29.72 ± 107.47 | | 98.17 ± 299.01 | | 44.23 ± 78.87 | | 12.57 ± 87.72 | | T | | 3 | | 3.228 | | 0.358 | |  |
|  |  |  | |  | |  | |  | | R | | 2 | | 0.115 | | 0.944 | |  |
|  |  |  | |  | |  | |  | | T*R | | 6 | | 9.230 | | 0.161 | |  |
| Benzyl nitrile | 84 | 29.56 ± 66.4 | | 37.76 ± 121 | | 87.49 ± 104.24 | | 28.26 ± 47.06 | | T | | 3 | | 2.806 | | 0.423 | |  |
|  |  |  | |  | |  | |  | | R | | 2 | | 1.341 | | 0.511 | |  |
|  |  |  | |  | |  | |  | | T*R | | 6 | | 2.518 | | 0.866 | |  |
| Methyl salicylate | 84 | 2.91 ± 25.14 | | -4.94 ± 23.12 | | 1.63 ± 29.24 | | 6.09 ± 24.83 | | T | | 3 | | 4.313 | | 0.230 | |  |
|  |  |  | |  | |  | |  | | R | | 2 | | 3.226 | | 0.199 | |  |
|  |  |  | |  | |  | |  | | T*R | | 6 | | 7.489 | | 0.278 | |  |
| 2-Aminobenzaldehyde | 84 | **139.17 ± 964.31** | | **199.47 ± 547.06** | | **284.45 ± 334.38** | | **116.09 ± 504.58** | | **T** | | **3** | | **12.618** | | **0.006** | |  |
|  |  | A | | B | | AB | | AB | | R | | 2 | | 0.394 | | 0.821 | |  |
|  |  |  | |  | |  | |  | | T*R | | 6 | | 14.371 | | 0.026 | |  |
| *p*-Anisaldehyde | 84 | 4.99 ± 23.79 | | 2.31 ± 37.22 | | 10.27 ± 37.56 | | 2.28 ± 23.06 | | T | | 3 | | 2.684 | | 0.443 | |  |
|  |  |  | |  | |  | |  | | R | | 2 | | 0.874 | | 0.646 | |  |
|  |  |  | |  | |  | |  | | T*R | | 6 | | 5.673 | | 0.461 | |  |
| Indole | 84 | 54.04 ± 152.61 | | 35.97 ± 129.85 | | 97.29 ± 124.61 | | 60.28 ± 134.02 | | T | | 3 | | 3.290 | | 0.349 | |  |
|  |  |  | |  | |  | |  | | R | | 2 | | 1.501 | | 0.472 | |  |
|  |  |  | |  | |  | |  | | T*R | | 6 | | 4.029 | | 0.673 | |  |
| Methyl anthranilate | 84 | 90.68 ± 354.5 | | -153.97 ± 1239.93 | | 103.68 ± 161.7 | | 25.33 ± 157.82 | | T | | 3 | | 0.723 | | 0.868 | |  |
|  |  |  | |  | |  | |  | | R | | 2 | | 0.541 | | 0.763 | |  |
|  |  |  | |  | |  | |  | | T*R | | 6 | | 0.969 | | 0.987 | |  |
| (*E,E*)-α-Farnesene | 84 | 131.63 ± 834.24 | | 107.12 ± 639.68 | | 512.79 ± 693.79 | | 514.26 ± 822.68 | | T | | 3 | | 1.450 | | 0.694 | |  |
|  |  |  | |  | |  | |  | | R | | 2 | | 1.228 | | 0.541 | |  |
|  |  |  | |  | |  | |  | | T*R | | 6 | | 7.378 | | 0.287 | |  |

**Table S3**. Results of the linear mixed models to test the effect of pollination, herbivory and the interaction of P*H on the sibling reaction norms. The discriminant functions obtained from linear discriminant analyses performed with (i) only the glucosinolates (Fig. 3A), (ii) only the floral traits (floral morphometry and volatiles, Fig. 3B), and (iii) with the leaf glucosinolates and floral traits combined (Fig. 3C). The mixed models included pollination, herbivory and P*H as fixed factors, and ‘replicate’ as random factor.

| Traits included in LDA | Linear discriminant | *N* | Factor | df | *F* | *P* |
| --- | --- | --- | --- | --- | --- | --- |
| (i) Only leaf glucosinolates (9 glucosinolates) | Linear discriminant 1 | 93 | Pollination (P) | 1 | 1.4257 | 0.2357 |
|  |  |  | Herbivory (H) | 1 | 3.3075 | 0.0724 |
|  |  |  | **P*H** | **1** | **8.5084** | **0.0045** |
|  | Linear discriminant 2 | 93 | **Pollination (P)** | **1** | **4.51** | **0.0365** |
|  |  |  | Herbivory (H) | 1 | 0.547 | 0.4615 |
|  |  |  | P*H | 1 | 0.227 | 0.6349 |
|  | Linear discriminant 3 | 93 | Pollination (P) | 1 | 0.0122 | 0.9121 |
|  |  |  | Herbivory (H) | 1 | 0.4085 | 0.5244 |
|  |  |  | P*H | 1 | 0.2687 | 0.6055 |
| (ii) Only floral traits (19 traits) | Linear discriminant 1 | 78 | **Pollination (P)** | **1** | **47.1844** | **<.0001** |
|  |  |  | Herbivory (H) | 1 | 0.1582 | 0.699 |
|  |  |  | **P*H** | **1** | **23.2676** | **<.0001** |
|  | Linear discriminant 2 | 78 | Pollination (P) | 1 | 2.0806 | 0.1535 |
|  |  |  | **Herbivory (H)** | **1** | **25.7005** | **<.0001** |
|  |  |  | P*H | 1 | 0.3965 | 0.5309 |
|  | Linear discriminant 3 | 78 | **Pollination (P)** | **1** | **6.4438** | **0.0133** |
|  |  |  | Herbivory (H) | 1 | 1.1104 | 0.2955 |
|  |  |  | **P*H** | **1** | **14.7605** | **0.0003** |
| (iii) Leaf glucosinolates, floral morphometry and floral volatiles (28 traits) | Linear discriminant 1 | 45 | **Pollination (P)** | **1** | **31.455** | **<.0001** |
|  |  |  | Herbivory (H) | 1 | 1.8526 | 0.1812 |
|  |  |  | **P*H** | **1** | **158.594** | **<.0001** |
|  | Linear discriminant 2 | 45 | **Pollination (P)** | **1** | **50.5618** | **<.0001** |
|  |  |  | **Herbivory (H)** | **1** | **7.1908** | **0.0107** |
|  |  |  | **P*H** | **1** | **8.0959** | **0.007** |
|  | Linear discriminant 3 | 45 | Pollination (P) | 1 | 1.2145 | 0.277 |
|  |  |  | **Herbivory (H)** | **1** | **37.212** | **<.0001** |
|  |  |  | P*H | 1 | 2.2041 | 0.1453 |

**Table S4**. Results of the linear mixed models to test the effect of pollination, herbivory and the interaction of P*H on the sibling reaction norm. Linear mixed models were performed with the sibling reaction norm calculated as the difference of noninfested minus infested values per sibling pairs. LMMs were done with each single trait as response variable, including Pollination, Herbivory and P*H as fixed factors, and ‘replicate’ as random factor. Values in bold indicate a significant effect (*P*<0.05).

| Trait | *N* | Factor | df | *F* | *P* |
| --- | --- | --- | --- | --- | --- |
| **Leaf glucosinolates** |  |  |  |  |  |
| Glucobrassicanapin | 93 | Pollination (P) | 1 | 2.5109 | 0.1167 |
|  |  | Herbivory (H) | 1 | 0.9578 | 0.3304 |
|  |  | PxH | 1 | 0.9082 | 0.3432 |
| Glucoerucin | 93 | Pollination (P) | 1 | 0.368 | 0.5457 |
|  |  | Herbivory (H) | 1 | 0.336 | 0.5636 |
|  |  | PxH | 1 | 0.1252 | 0.7243 |
| Gluconapin | 93 | Pollination (P) | 1 | 1.2873 | 0.2597 |
|  |  | Herbivory (H) | 1 | 1.0576 | 0.3066 |
|  |  | PxH | 1 | 1.1037 | 0.2963 |
| Gluconasturtiin | 93 | Pollination (P) | 1 | 2.7117 | 0.1032 |
|  |  | Herbivory (H) | 1 | 0.151 | 0.6985 |
|  |  | PxH | 1 | 0.2677 | 0.6062 |
| Glucoraphanin | 93 | Pollination (P) | 1 | 0.0124 | 0.9115 |
|  |  | Herbivory (H) | 1 | 0.0993 | 0.7534 |
|  |  | PxH | 1 | 0.1386 | 0.7106 |
| Glucobrassicin | 93 | Pollination (P) | 1 | 0.3184 | 0.574 |
|  |  | Herbivory (H) | 1 | 0.7776 | 0.3803 |
|  |  | PxH | 1 | 3.1783 | 0.0781 |
| 4-Hydroxyglucobrassicin | 93 | Pollination (P) | 1 | 0.0568 | 0.8121 |
|  |  | Herbivory (H) | 1 | 1.703 | 0.1953 |
|  |  | **PxH** | **1** | **4.4396** | **0.038** |
| 4-Methoxyglucobrassicin | 93 | Pollination (P) | 1 | 1.4721 | 0.2282 |
|  |  | Herbivory (H) | 1 | 0.3342 | 0.5647 |
|  |  | PxH | 1 | 2.0358 | 0.1571 |
| Neoglucobrassicin | 93 | Pollination (P) | 1 | 0.2831 | 0.596 |
|  |  | Herbivory (H) | 1 | 0.0011 | 0.9736 |
|  |  | PxH | 1 | 0.3619 | 0.549 |
| **Flower morphometry** | |  |  |  |  |
| Sepal length | 149 | Pollination (P) | 1 | 1.9426 | 0.1656 |
|  |  | Herbivory (H) | 1 | 0.4599 | 0.4988 |
|  |  | PxH | 1 | 1.0637 | 0.3041 |
| Petal length | 149 | Pollination (P) | 1 | 0.0617 | 0.8042 |
|  |  | Herbivory (H) | 1 | 2.4158 | 0.1223 |
|  |  | **PxH** | **1** | **6.096** | **0.0147** |
| Petal width | 149 | Pollination (P) | 1 | 1.1118 | 0.2935 |
|  |  | Herbivory (H) | 1 | 0.2162 | 0.6426 |
|  |  | PxH | 1 | 3.1965 | 0.0759 |
| Pistil length | 149 | Pollination (P) | 1 | 0.6511 | 0.421 |
|  |  | Herbivory (H) | 1 | 1.3636 | 0.2449 |
|  |  | PxH | 1 | 3.668 | 0.0575 |
| Large stamen | 149 | Pollination (P) | 1 | 1.1448 | 0.2864 |
|  |  | Herbivory (H) | 1 | 0.5292 | 0.4681 |
|  |  | PxH | 1 | 2.152 | 0.1446 |
| Short stamen | 149 | Pollination (P) | 1 | 0.0263 | 0.8715 |
|  |  | Herbivory (H) | 1 | 0.4213 | 0.5173 |
|  |  | PxH | 1 | 0.127 | 0.722 |
| **Flower volatiles** |  |  |  |  |  |
| Benzaldehyde | 84 | Pollination (P) | 1 | 0.185 | 0.3702 |
|  |  | Herbivory (H) | 1 | 0.0366 | 0.4822 |
|  |  | PxH | 1 | 0.6688 | 0.4587 |
| 1-Butene-4-isothyocyanate | 84 | Pollination (P) | 1 | 1.3417 | 0.2502 |
|  |  | Herbivory (H) | 1 | 3.2311 | 0.0761 |
|  |  | PxH | 1 | 0.825 | 0.3665 |
| 6-Methyl-5-hepten-2-one | 84 | Pollination (P) | 1 | 0.9938 | 0.3218 |
|  |  | Herbivory (H) | 1 | 0.0832 | 0.7738 |
|  |  | PxH | 1 | 0.0457 | 0.8313 |
| *(Z)*-3-Hexenyl acetate | 84 | Pollination (P) | 1 | 1.7468 | 0.1901 |
|  |  | Herbivory (H) | 1 | 1.8251 | 0.1806 |
|  |  | PxH | 1 | 3.4238 | 0.068 |
| Phenylacetaldehyde | 84 | **Pollination (P)** | **1** | **5.3543** | **0.0233** |
|  |  | Herbivory (H) | 1 | 0.264 | 0.6088 |
|  |  | PxH | 1 | 0.677 | 0.4131 |
| Methyl benzoate | 84 | Pollination (P) | 1 | 0.6873 | 0.4096 |
|  |  | Herbivory (H) | 1 | 0.2434 | 0.6231 |
|  |  | PxH | 1 | 1.7087 | 0.195 |
| Benzyl nitrile | 84 | **Pollination (P)** | **1** | **5.7758** | **0.0186** |
|  |  | Herbivory (H) | 1 | 0.3386 | 0.5623 |
|  |  | PxH | 1 | 0.156 | 0.6939 |
| Methyl salicylate | 84 | Pollination (P) | 1 | 0.0965 | 0.7568 |
|  |  | Herbivory (H) | 1 | 0.095 | 0.7587 |
|  |  | PxH | 1 | 0.0073 | 0.9323 |
| 2-Aminobenzaldehyde | 84 | Pollination (P) | 1 | 0.3932 | 0.5324 |
|  |  | Herbivory (H) | 1 | 0.3478 | 0.5571 |
|  |  | PxH | 1 | 0.301 | 0.5848 |
| *p*-Anisaldehyde | 84 | Pollination (P) | 1 | 0.0004 | 0.9837 |
|  |  | Herbivory (H) | 1 | 0.3583 | 0.5512 |
|  |  | PxH | 1 | 0.1456 | 0.7038 |
| Indole | 84 | Pollination (P) | 1 | 1.3326 | 0.2518 |
|  |  | Herbivory (H) | 1 | 0.1324 | 0.7169 |
|  |  | PxH | 1 | 0.025 | 0.8749 |
| Methyl anthranilate | 84 | Pollination (P) | 1 | 0.4492 | 0.5047 |
|  |  | Herbivory (H) | 1 | 0.3308 | 0.5669 |
|  |  | PxH | 1 | 0.0867 | 0.7691 |
| (*E,E*)-α-Farnesene | 84 | Pollination (P) | 1 | 1.4127 | 0.2382 |
|  |  | Herbivory (H) | 1 | 0.0134 | 0.9081 |
|  |  | PxH | 1 | 0.1543 | 0.6956 |

**Table S5**. Univariate comparisons of 21 leaf volatiles from infested plants between treatments. Leaf volatiles (mean ± s.d.) were collected only from previously infested plants. Different letters indicate significant differences following Tukey *post-hoc* tests. Values in bold indicate significant factor effects in the LMMs (treatment, T; replicate, R; and the interaction of T*R). Volatile units are pg^-1^ plant^-1^ L . Treatments: H_H_– = hand-pollination and no herbivory; H_H_+, hand-pollination and constant herbivory; B_H_+, bee-pollination and constant herbivory, B_H_–, bee-pollination and no herbivory.

| Leaf volatile | *N* | H_H_– | H_H+_ | B_H+_ | B_H_– | Source | df | *F* | *P* |
| --- | --- | --- | --- | --- | --- | --- | --- | --- | --- |
| alpha-Pinene | 174 | 0.025 ± 0.013 | 0.042 ± 0.037 | 0.035 ± 0.029 | 0.024 ± 0.017 | T | 3 | 2.4816 | 0.063 |
|  |  |  |  |  |  | R | 2 | 0.6873 | 0.504 |
|  |  |  |  |  |  | **T*R** | **6** | **2.9575** | **0.009** |
| Benzaldehyde | 174 | 0.065 ± 0.04 | 0.073 ± 0.067 | 0.059 ± 0.044 | 0.072 ± 0.048 | T | 3 | 1.2457 | 0.295 |
|  |  |  |  |  |  | R | 2 | 15.3407 | **0.001** |
|  |  |  |  |  |  | T*R | 6 | 1.0106 | 0.420 |
| 1,3,5-trimethyl-Benzene | 174 | 0.094 ± 0.053 | 0.099 ± 0.043 | 0.092 ± 0.044 | 0.102 ± 0.051 | T | 3 | 1.4101 | 0.241 |
|  |  |  |  |  |  | **R** | **2** | **24.8098** | **0.001** |
|  |  |  |  |  |  | T*R | 6 | 0.9633 | 0.452 |
| beta-Pinene | 174 | 0.012 ± 0.006 | 0.017 ± 0.012 | 0.015 ± 0.01 | 0.011 ± 0.006 | T | 3 | 2.2631 | 0.083 |
|  |  |  |  |  |  | R | 2 | 0.8736 | 0.419 |
|  |  |  |  |  |  | **T*R** | **6** | **2.5978** | **0.020** |
| 1-Butene-4-isothiocyanate | 174 | 0.14 ± 0.109 | 0.1 ± 0.093 | 0.136 ± 0.142 | 0.178 ± 0.154 | T | 3 | 1.5875 | 0.195 |
|  |  |  |  |  |  | R | 2 | 1.5985 | 0.205 |
|  |  |  |  |  |  | T*R | 6 | 1.0382 | 0.403 |
| Limonene | 174 | 0.209 ± 0.109 | 0.22 ± 0.096 | 0.216 ± 0.102 | 0.207 ± 0.085 | T | 3 | 1.0225 | 0.384 |
|  |  |  |  |  |  | **R** | **2** | **45.5874** | **<.0001** |
|  |  |  |  |  |  | T*R | 6 | 1.6344 | 0.141 |
| Acetophenone | 174 | 0.045 ± 0.038 | 0.07 ± 0.069 | 0.07 ± 0.105 | 0.063 ± 0.087 | T | 3 | 2.0519 | 0.109 |
|  |  |  |  |  |  | **R** | **2** | **6.0674** | **0.003** |
|  |  |  |  |  |  | T*R | 6 | 0.6772 | 0.668 |
| Undecane | 174 | 0.024 ± 0.014 | 0.028 ± 0.011 | 0.025 ± 0.008 | 0.028 ± 0.018 | T | 3 | 2.1493 | 0.096 |
|  |  |  |  |  |  | R | 2 | 1.1864 | 0.308 |
|  |  |  |  |  |  | T*R | 6 | 0.6695 | 0.674 |
| Linalool | 174 | **0.054 ± 0.058** | **0.024 ± 0.022** | **0.02 ± 0.025** | **0.016 ± 0.029** | **T** | **3** | **6.1384** | **0.001** |
|  |  | **A** | **AB** | **B** | **B** | R | 2 | 0.6969 | 0.500 |
|  |  |  |  |  |  | T*R | 6 | 0.58 | 0.746 |
| Camphor | 174 | 0.012 ± 0.003 | 0.013 ± 0.003 | 0.012 ± 0.002 | 0.012 ± 0.002 | T | 3 | 0.7082 | 0.548 |
|  |  |  |  |  |  | R | 2 | 2.8865 | 0.059 |
|  |  |  |  |  |  | T*R | 6 | 2.0505 | 0.062 |
| Menthol | 174 | 0.00023 ± 0.0002 | 0.00021 ± 0.00012 | 0.00026 ± 0.00018 | 0.00027 ± 0.00037 | T | 3 | 2.2467 | 0.085 |
|  |  |  |  |  |  | R | 2 | 0.7985 | 0.452 |
|  |  |  |  |  |  | T*R | 6 | 1.8385 | 0.095 |
| Methyl salicylate | 174 | 0.001 ± 0.003 | 0.001 ± 0.001 | 0.002 ± 0.002 | 0.001 ± 0.002 | T | 3 | 2.6342 | 0.052 |
|  |  |  |  |  |  | R | 2 | 4.4519 | 0.013 |
|  |  |  |  |  |  | **T*R** | **6** | **2.487** | **0.025** |
| Benzothiazole | 174 | 0.022 ± 0.012 | 0.025 ± 0.016 | 0.026 ± 0.015 | 0.023 ± 0.009 | T | 3 | 2.1825 | 0.092 |
|  |  |  |  |  |  | R | 2 | 1.348 | 0.263 |
|  |  |  |  |  |  | T*R | 6 | 1.4078 | 0.215 |
| Indole | 174 | 0.00027 ± 0.0002 | 0.00031 ± 0.00023 | 0.00029 ± 0.00021 | 0.00028 ± 0.00023 | T | 3 | 1.8123 | 0.147 |
|  |  |  |  |  |  | **R** | **2** | **6.0608** | **0.003** |
|  |  |  |  |  |  | T*R | 6 | 1.212 | 0.303 |
| Tridecane | 174 | 0.011 ± 0.005 | 0.013 ± 0.01 | 0.011 ± 0.008 | 0.012 ± 0.007 | T | 3 | 0.6406 | 0.590 |
|  |  |  |  |  |  | **R** | **2** | **15.5198** | **<.0001** |
|  |  |  |  |  |  | T*R | 6 | 0.5149 | 0.797 |
| Formanilide | 174 | 0.003 ± 0.002 | 0.002 ± 0.002 | 0.003 ± 0.004 | 0.02 ± 0.077 | T | 3 | 0.3136 | 0.816 |
|  |  |  |  |  |  | R | 2 | 8.2184 | <0.001 |
|  |  |  |  |  |  | T*R | 6 | 1.6142 | 0.146 |
| alpha-Cedrene | 174 | 0.003 ± 0.002 | 0.003 ± 0.001 | 0.002 ± 0.001 | 0.003 ± 0.002 | T | 3 | 1.4392 | 0.233 |
|  |  |  |  |  |  | R | 2 | 18.091 | <0.001 |
|  |  |  |  |  |  | T*R | 6 | 1.3932 | 0.220 |
| 2,2,6-Trimethyl-bicyclo[4.1.0]hept-1-yl-methanol | 174 | 0.0039 ± 0.0035 | 0.004 ± 0.0024 | 0.0036 ± 0.002 | 0.0042 ± 0.0032 | T | 3 | 1.8851 | 0.134 |
|  |  |  |  |  |  | R | 2 | 14.8487 | <0.001 |
|  |  |  |  |  |  | T*R | 6 | 1.5536 | 0.164 |
| 11,13.Dimethyl-12-tetradecen-1-ol,acetate | 174 | 0.003 ± 0.002 | 0.003 ± 0.002 | 0.003 ± 0.002 | 0.003 ± 0.002 | T | 3 | 1.0009 | 0.394 |
|  |  |  |  |  |  | R | 2 | 6.4624 | 0.002 |
|  |  |  |  |  |  | T*R | 6 | 0.95 | 0.461 |
| Tetradecane | 174 | 0.022 ± 0.014 | 0.031 ± 0.045 | 0.026 ± 0.037 | 0.024 ± 0.008 | T | 3 | 1.1681 | 0.324 |
|  |  |  |  |  |  | **R** | **2** | **3.2712** | **0.040** |
|  |  |  |  |  |  | T*R | 6 | 0.1918 | 0.979 |
| Geranyl acetone | 174 | 0.008 ± 0.006 | 0.007 ± 0.003 | 0.006 ± 0.002 | 0.007 ± 0.003 | T | 3 | 1.5441 | 0.205 |
|  |  |  |  |  |  | R | 2 | 1.4964 | 0.227 |
|  |  |  |  |  |  | T*R | 6 | 1.6016 | 0.150 |
| Total VOCs | 174 | 0.008 ± 0.006 | 0.007 ± 0.003 | 0.006 ± 0.002 | 0.007 ± 0.003 | T | 3 | 0.9583 | 0.414 |
|  |  |  |  |  |  | **R** | **2** | **3.1711** | **0.045** |
|  |  |  |  |  |  | T*R | 6 | 1.1451 | 0.339 |
